# Combination of density functional theory and calorimetry reveals the microscopic nature of spin state switching in 1D Fe(ii) spin crossover complexes†

**DOI:** 10.1039/d5ra03472h

**Published:** 2025-09-09

**Authors:** Juliusz A. Wolny, Xiaochun Li, Marinela Dîrtu, Konstantin Gröpl, Tim Hochdörffer, Hauke Paulsen, Yann Garcia, Volker Schünemann

**Affiliations:** a Department of Physics, University of Kaiserslautern-Landau Erwin-Schrödinger-Str. 46 67663 Kaiserslautern Germany wolny@rptu.de; b Institute of Condensed Matter and Nanosciences, Molecular Chemistry, Materials and Catalysis (IMCN/MOST), Université catholique de Louvain Louvain-la-Neuve 1348 Belgium; c Institut für Physik, Universität zu Lübeck Ratzeburger Allee 160, D-23562 Lübeck Germany

## Abstract

The Gibbs free energy of spin transitions in the heptanuclear models of 1D Fe(ii) spin crossover 1,2,4-triazole complexes has been estimated using DFT methods. The complexes modelled were [Fe(Htrz)_2_trz]BF_4_ (Htrz = 4*H*-1,2,4-triazole, trz = 1,2,4-triazolato), 1 the dehydrated and hydrated [Fe(NH_2_trz)_3_]Cl_2_ (NH_2_trz = 4-amino-1,2,4-triazole), 2 and 2a, and [Fe(NH_2_trz)_3_](NO_3_)_2_, 3. For each complex, the electronic energy and the vibrational energies were calculated for a heptanuclear model containing five inner Fe(ii) centres in the high-spin (HS) and the low-spin (LS) states. All other possible 18 spin isomers with one to four HS centres were also modelled. Results obtained using different exchange–correlation functionals based on the B3LYP one show that each spin isomer with a particular permutation of HS and LS centres within the pentanuclear linear unit has distinctive electronic and vibrational energies. The electronic energy of each spin isomer was found to be equal to the sum of the adiabatic electronic energy of the spin transition *E*_ad_ given by the difference in energies between the LS and HS states and the strain energy *H*_strain_. This quantity is non-zero for any spin isomer containing both LS and HS centres. Unlike *E*_ad_, which has also been determined experimentally by calorimetric measurements, *H*_strain_ is independent of the applied functional. Calculations of the temperature dependence of the Gibbs free energy change Δ*G* of 19 possible spin transitions for heptanuclear model systems reveals that strain effects lead to an additional destabilisation of the spin isomers containing both LS and HS centres. The actual strain pattern depends on the chemical structure of the model molecule.

## Abstract 2

DFT supported by calorimetry show that the spin transition energies in 1D chains are the sum of the electronic transition energy and the strain due to elastic effects. The thermodynamics of the spin transitions in the chains were also calculated.

## Graphical abstract

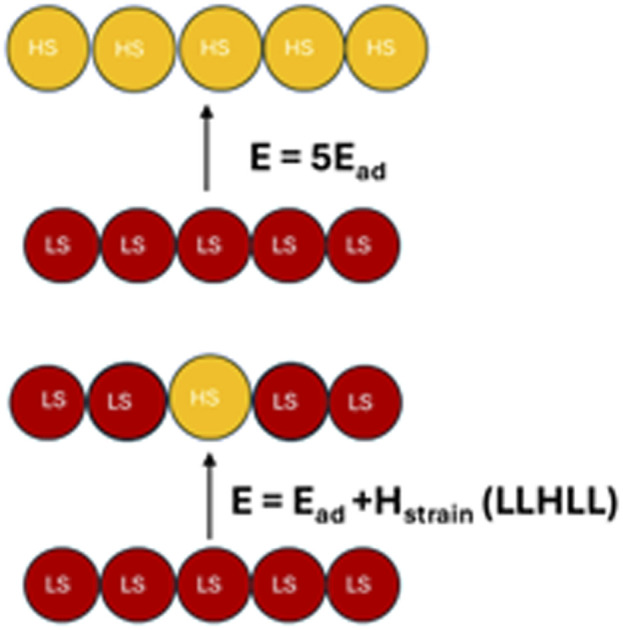

## Introduction

Dynamic lattice properties lie at the heart of the spin crossover (SCO) phenomenon.^1^ For a molecular crystal, the low energy acoustic phonons show movements of the molecules as a whole, while the optical phonons are characterized by more localized molecular vibrations that occur at higher energies. Phonons determine the key properties such as the heat capacity, vibrational entropy and energy, lattice rigidity, elastic constants and elastic interactions.^2^ All static^3^ or dynamical^4^ phenomenological models aiming to explain the cooperativity of spin transition and its spatiotemporal behaviour consider these magnitudes in more or less explicit form. As Palii, Klokishner *et al.*^5^ state, two general trends in modelling of cooperative spin transitions can be identified:

(i) The macroscopic approach focuses on the examination of elastic interactions that change when the spins switch. These interactions result in the coupling of spin centres as they are transmitted to the lattice. One of the most effective approaches is the mechano-elastic approach, which utilises molecular dynamics (MD) and Monte Carlo (MC) methods.^6^

(ii) The microscopic approach concerns the molecular electronic structure and normal modes, while the coupling between the electronic states of the SCO centres is transmitted by phonons.

In the light of the evolution of first-principle modelling of SCO materials over the last two decades, particularly the development of density functional theory (DFT)^7^ including functional performance^8^ and modelling of structural-spin transition relationships^9^ a pertinent question emerges: how can quantum chemistry provide links between the molecular properties of the SCO centre and the relevant parameters of microscopic and macroscopic models? For example, what is the relationship between the structural, electronic and vibrational properties of the high-spin (HS) and low-spin (LS) isomers of an SCO molecule and the elastic tensor components of the macroscopic model, or the coupling parameters of the microscopic model like that used in ref. 5. This is particularly relevant in the context of molecular material science, where a key problem is determining how the chemical composition of molecules influences the mechanical properties of the material from which they are composed.

In principle, quantum state calculations for solids (periodic calculations) should result in optimised geometries, cell volumes, electronic states, energies and vibrations. This would provide a thorough thermodynamic characterization of phases with different proportions of HS centres (*x*_HS_) and distinct spin centre distributions. According to a recent review the vast majority of calculations relating to the thermal dependence of the elastic tensor components have been conducted on simple organic molecules.^10^ However, the work of Erba *et al.*^11^ demonstrates that a quasi-harmonic method could provide a computationally affordable approach. The parameters and their temperature dependence, as determined by X-ray methods^12^ were utilised to estimate the direct interaction and self-energy contributions to the cooperativity parameter of Spiering's model.^13^ Thus, in principle, periodic calculations that yield magnitudes relevant to phenomenological models for a SCO complex are feasible. However, it must be noted that such calculations are hindered by two issues. The primary issue relates to the calculation of spin transition energy (see ref. 14 for the most recent review). The electronic energy of the spin transition, as calculated by DFT methods, depends on the exchange–correlation functional employed,^15^ even in the case of isolated molecules. This issue can be addressed in two ways. Firstly, a series of related systems can be compared, differing in terms of substituents or solvated molecules. Secondly, the experimental values of spin transition energies can be considered (ref. 14 and *vide infra*). The first approach has also been used for periodic calculations involving complexes with different anions^16^ and solvated molecules.^17^ Importantly, when considering different spatial combinations of a given number of HS and LS molecules, the dependency ceases to exist, while systems of the same multiplicity are modelled. In their study, Vela and Paulsen considered the approach of considering the different spatial distribution of HS and LS centres to ascertain the energy contribution of cooperativity for [Fe(2-pic)_3_]Cl_2_ (2-pic = 2-picolylamine)^18^ and the phenomenological interaction parameter *Γ* for [Fe(phen)_2_(NCS)_2_].^19^ Additionally, modelling of the SCO in the 3D Hoffman-clathrate [Fe^II^(TPB){Au^I^(CN)_2_}]I·4H_2_O·4DMF (TPB = 1,2,4,5-tetra(pyridin-4-yl)benzene)^20^ was performed by calculating electronic energies of HS or LS “spin defects” by introducing one LS or one HS centre in a supercell containing eight SCO centres. For periodic energy calculations, the Hubbard-like parameter *U* is typically used to describe intra-atomic Coulomb repulsions. This is done within the framework of the DFT + *U* approach, which introduces an additional energy term, *U*, for the localised electrons, while describing the delocalised electrons using DFT functionals alone.^21–23^

Another issue is the calculation of the vibrational modes, which determine the vibrational entropy that drives the SCO process. In this case, there is an inevitable conflict between achieving precise calculations and reducing computational time. It has been demonstrated that the latter increases in proportion to the size of the modelled molecules and the level of DFT tools utilised, as well as the algorithm employed for calculating Hessian matrix elements. The initial papers on periodic calculations of SCO complexes illustrate this. While modelling of the CsFe^II^[Cr^III^(CN)_6_] Prussian blue analogue^24^ yielded only three small imaginary frequencies (note that the electronic energies for this system were previously calculated with the GGA + *U* approach^25^ using the finite-differences method to assess the Hessian matrix elements), a paper reporting a similar approach to the [Fe(phen)_2_(NCS)_2_] complex mentions the general problem of imaginary frequencies related to a number of soft transitions below 50 cm^−1^.^26^ Therefore, achieving accuracy is likely to be more challenging for molecular crystals due to their weaker intermolecular interactions compared to ionic crystals. In a subsequent report Zhang^27^ calculated the electronic energy of spin transitions of molecular crystals of the Schiff-base ligand complex with an FeN_4_O_2_ coordination core. This study employed the LDA/GGA functionals within the DFT + *U* approach using Quantum-ESPRESSO (QE) package.^28^ SIESTA^29^ was used to calculate the harmonic vibrations using the finite-differences approximation (see ref. 30, where the accuracy and potential perspectives of this method are discussed). Subsequently, Vela *et al.*^22^ employed the same approach with QE for several additional SCO molecular crystals of Fe(ii). They indicated that computing both analytical frequencies and frequencies obtained with the finite-difference approximation at the crystalline phase would be prohibitive. To assess the vibrational entropy, the authors used values calculated for single molecules with functionals that go beyond the LDA/GGA. This approach was further developed in subsequent ref. 17–19. Collet *et al.*^31^ reported the use of terahertz, infrared and Raman spectroscopy on HS [Fe(phen)_2_(NCS)_2_] crystals, together with periodic calculations (GGA) using the VASP package.^32^ Calculating vibrations using the frozen-phonon method yielded reasonable energies in the low-frequency region of 20–270 cm^−1^. Recently, Poloni, Rodríguez-Velamazán *et al.*^33^ reported PBE + D2 calculations of the phonon density of states (pDOS) with QE for the Fe(pz)[Pt(CN)_4_] (pz = pyrazine) 3D SCO complex and its SO_2_ adduct, obtaining a fit to inelastic neutron scattering (INS) data. Harmonic interatomic force constants were computed using density functional perturbation theory.^34^ Recently Wu and coworkers^35^ demonstrated that the PBE + U3 + D3 approach for periodic calculations allows the reproducing of the spin-transition temperatures in a series of 2D complexes. In summary, despite recent advances in periodic DFT calculations for SCO materials, these calculations still require substantial computing time and cannot be used with functionals beyond the GGA approximation. This has implications for the accuracy of low-frequency phonon mode energies, particularly in the context of molecular crystals.

Consequently, modelling the thermodynamics of multiple spatial distributions of spin centres for various spin isomers is an alternative approach for the time being. Another possibility is to use MD to model crystals involving millions of atoms.^36^ The quality of the calculations can be improved by using DFT calculated force constants. This has resulted in a favourable alignment between the experimental and MD calculated pDOS for models of the 1D Fe(ii) polynuclear SCO chains based on 1,2,4-triazole-type ligands.^37^ In a recent report the Bousseksou group demonstrated that MD calculations (with no DFT calibration of the force constants) for the crystals of the 3D SCO compound Fe(pz)[Ni(CN)_4_] successfully reproduced the thermodynamic and mechanical parameters, as well as the pDOS of both, the LS and HS phases.^38^ Importantly, this approach enabled surface effects to be estimated, which are essential for explaining the hysteretic behaviour of the spin transition.^13^ MD has been combined with the MC approach^39,40^ or with MC and ligand field molecular mechanics^41^ to model the spin transition energy and mechanisms in the above mentioned Fe(pz)[Pt(CN)_4_] 3D SCO complex. It is reasonable to assume that this approach will provide superior modelling of the soft phonons if the lattice under investigation comprises covalently bound units rather than being a molecular crystal.

The anharmonicity of vibrations restricts the accuracy of the harmonic approximation, especially regarding the thermal lattice expansion of the system and its thermoelasticity.^42^ Another issue is that the harmonic approximation neglects intrinsic phonon anharmonicity and phonon–phonon coupling, which has consequences for the computed thermodynamic properties. These problems can be overcome using the quasi-harmonic approximation (QHA).^42^ Alternatively, optimised scale factors can be applied when calculating vibrational, harmonic and fundamental frequencies, as well as zero-point energies (ZPE).^43^ Additionally, a composite scheme has been proposed that considers anharmonicity when calculating molecular entropy by combining DFT, semi-empirical and force-field approximations.^44^

In this context, it is noteworthy that calculations of anharmonic effects for spin crossover systems are feasible with commonly used software packages, despite being computationally much more demanding than calculations of harmonic frequencies.^45^ In this work we neglect anharmonic effects according to the following quotation of ref. 45: “as a conclusion, we put forward that for high precision results, one should be aware of the anharmonic effects, but as long as computational chemistry is still struggling with other larger factors like the influence of the environment and the accurate determination of the electronic energy difference between HS and LS, the anharmonicity of the vibrational modes is a minor concern”. Conversely, accounting for anharmonic effects is essential for phenomenological models of elasticity in SCO systems.^13,46^

This presentation outlines the results of DFT modelling of SCO assemblies with various spin-state permutations. This approach sheds light on the differences in thermodynamic and vibrational parameters between phases with different distributions of HS and LS centres. We used 1,2,4-triazole derivatives as model compounds to simulate one-dimensional (1D) systems involving pentanuclear, hexanuclear and nonanuclear chains.^47^ This enabled us to achieve a satisfactory correlation between the ^57^Fe-pDOS derived theoretically and that obtained experimentally by nuclear inelastic scattering (NIS).^48^ Furthermore, modelling 3D Fe(pz)[Pt(CN)_4_] with a cubic cell containing 15 SCO centres yielded good agreement between the calculated ^57^Fe-pDOS and the experimental pDOS obtained from nuclear inelastic scattering (NIS) measurements.^49^ We also present calculations of the temperature dependence of the entropy difference Δ*S* and the Gibbs free energy difference Δ*G* of the spin transition for different spin isomers.

To quantify intramolecular cooperativity, we have introduced the parameter *H*_coop_, defined as the difference of the LS → HS spin transition electronic energy of a SCO centre with the neighbours being in LS (L) and HS (H) state,^50,47*d*^*i.e.* between LLLLL → LLHLL and HHLHH → HHHHH transitions energies. *H*_coop_ was shown to be essentially independent on the applied functional. In this report we introduce a novel parameter, *H*_strain_, which quantifies the strain present in each possible spin isomer and show that *H*_coop_ contains this quantity for two particular spin isomers. A related parameter, *H*_block_, was recently introduced, which can be used to determine the cooperativity of the spin transition in mononuclear complexes based on the electronic energies of the LS and HS isomers within matrices that have been optimised using periodic calculations for a given spin of the switching centres.^51^ To quote the authors “*H*_block_ quantifies the structural quenching of a SCO molecule to remain in a given spin-state”.

The obvious cost of modelling the linear 1D SCO chain materials with a single chain of finite size is that the hysteretic character of the spin transition cannot be predicted since it is related to long-range interactions.^13^ In other words, the sharp transitions in such systems take place in 2D and 3D.^52^ Indeed, the macroscopic mean-field approach reveals that the first-order (sharp and hysteretic) transitions occur when both, the short-range (intrachain) and long-range (interchain) interactions are ferroelastic (*i.e.* the pure LS and HS phases are thermodynamically preferred). The two-step transitions occur with antiferroelastic (*i.e.* the alternating LHLHLH-like phases are thermodynamically preferred) short-range interactions and ferroelastic long-range interactions. The importance of short-range ferroelastic interactions for the first-order spin transition has been recognized.^53^ Although the modelling of 1D SCO chains cannot account for the long-range (interchain) interactions, one may expect a fairly accurate modelling of the short-range intrachain interactions. Hence, such simulations could predict which of the following scenarios will occur for a given 1D SCO chain:

(a) The pure LS and HS phases correspond to the lowest Gibbs free energy as a function of temperature. This corresponds to the dominant ferroelastic short-range interactions and is a prerequisite for a sharp transition (enhanced by the ferroelastic long-range interactions).

(b) The alternate LHLHLH-like phase emerges as the new ground state at some temperature replacing the pure LS phase. This corresponds to the dominant antiferroelastic short-range interactions and is a prerequisite for a two-step transition (again enhanced by the ferroelastic long-range interactions).

(c) None of the above takes place, the phases with different molar fractions of HS appear to have the lowest Gibbs free energy with increasing temperature. Neither elastic nor antiferroelastic interactions dominate and either a soft or an incomplete spin transition occurs.

The current report describes the results of the DFT modelling of the oligonuclear models of the 1D SCO chains of Fe(ii) complexes with 4*H*-R-1,2,4-triazole ligands, namely [Fe(Htrz)_2_trz]BF_4_ (Htrz = 4*H*-1,2,4-triazole, trz = 1,2,4-triazolato) (1), [Fe(NH_2_trz)_3_]Cl_2_ (NH_2_trz = 4-amino-1,2,4-triazole) (2), [Fe(NH_2_trz)_3_]Cl_2_·2.5H_2_O (2a) and [Fe(NH_2_trz)_3_](NO_3_)_2_ (3). These materials are one of the most extensively investigated SCO complexes due to their remarkable characteristics with thermal spin transitions often accompanied by wide hysteresis loops with transition temperatures located close to room temperature, leading to potential applications, *e.g.* as memory and sensor devices.^54^ Recent work has demonstrated the additional fascinating effects of such 1D coordination polymers, such as the dependence of the spin transition on particle size^55^ and mechanochemical recrystallisation,^56^ as well as pronounced lattice softening of 1 upon replacing some of the Htrz ligands with NH_2_trz.^57^ These molecules contain the rigid bridging 1,2,4-triazole ring that distorts the geometry of the neighbours of a given spin if a centre is of different spin.^47^ This effect leads to a particularly strong short-range interactions (see ref. 13) with a *Γ* value of more than 10^3^ cm^−1^ (ref. 58) for 1.^59^ Typically, in molecular crystals the direct elastic interactions between molecules does not exceed 10–100 cm^−1^.^13^ Our previous results for 1 and [Fe(NH_2_trz)_3_]X_2_ revealed that the DFT modelling of oligonuclear fragments provides a very good fit to the experimentally observed pDOS, both for the pure Fe(ii) complex and for the Zn(ii)-diluted ones.^41^

This report is organised as follows: firstly, we describe the modelling of four SCO materials – 1, 2, 2a (ref. 60 and 61) and 3.^62^ For each complex a heptanuclear model molecule was optimised with the B3LYP* functional. There are 20 different spin isomers with 5 switching Fe(ii) centres. For each the electronic energy and the normal vibrations were calculated. The latter gave the vibrational contribution to entropy, *S*_vib_. This allowed the calculations of the thermal dependence of the Gibbs free energy for all spin isomers. Further, we estimated the electronic energies of all spin isomers using other functionals (B3LYP and CAM-B3LYP) for the geometries obtained with B3LYP* or after optimisation. Then, for comparison, we repeated the optimisation and vibrational calculations for all systems using the B3LYP functional with dispersion correction. The analysis of the obtained electronic energies revealed that the relative (respective to that of the pure LS state) energy of each spin isomer is a sum of two factors: (a) functional dependent spin transition energy (b) strain energy due to presence of both LS and HS centres. The dependence of this energy on the applied functional is discussed. The temperature dependence of the Gibbs free energy for the B3LYP functional points towards ferroelastic interaction within the chain. The values of energy and entropy of the spin transition for 1, 2 and 2a were also determined experimentally by differential scanning calorimetry (DSC). The temperature dependence of Δ*G* was also derived based on the electronic spin transition energies derived from calorimetric measurements.

## Results and discussion

### Calculations of electronic energies and thermodynamic functions of different spin isomers of the heptanuclear models of 1, 2, 2a and 3

**Scheme 1:**
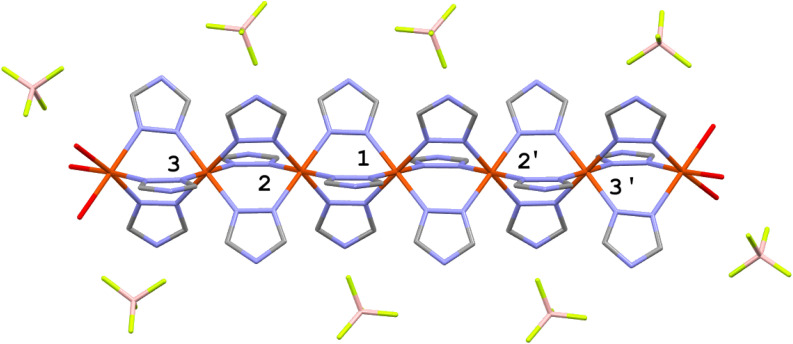
Graphic representation of the heptanuclear model used for calculations. The numbering of iron centres is given. The model molecule of 1 is shown.

The electronic energies of 1, 2, 2a and 3 were calculated using the models described in the Experimental and computational methods section. Following the approach described in ref. 47, heptanuclear models were used in which the five inner Fe-atoms were coordinated with six 1,2,4-triazole-type ligands, while the terminal atoms were coordinated with three bridging ligands and three water molecules and were kept in the HS state for all spin isomers. The model of 1 is shown in Scheme 1. The optimised coordinates of all 20 spin isomers for the systems under study are given in the SI. The most important obtained structural feature is the shortening of the Fe–N bonds of the HS centres by their LS neighbours and the lengthening of the Fe–N bonds of the LS centre caused be its HS neighbours. This effect was previously modelled with DFT and detected with nuclear inelastic scattering.^47^ It is also reflected in Table 1 which lists the mean Fe–N distances for the pure LS and HS models of the complexes under study compared to the spin isomers revealing the largest strain (*H*_strain_*vide infra*). For further information see the list of all obtained atomic coordinates in SI.

**Table 1:** Mean Fe–N values for the LLLLL and HHHHH isomers of the complexes under study compared to those isomers revealing the highest strain (*vide infra*). Note the increase of the LS Fe(2)–N with HS neighbours bonds by *ca.* 0.2 Å compared to LS-Fe(2) ones with LS neighbours for 1, 2 and 2a and that of LS Fe(1) for LHLHL isomer of 3 (the corresponding pairs given in bold). The decrease of the Fe–N bonds for the HS centre upon introduction of the LS neighbours for 1, 2 and 2a varies from 0.2 Å for 1 to 0.32 Å for 2a. This shortening is 0.21 Å for Fe(2′) in 3 (the corresponding pairs marked blue). The distances for the negatively charge trz^−^ ligand in 1 are given in italics

		Fe(3)	Fe(2)	Fe(1)	Fe(2′)	Fe(3′)
1	LLLLL	1.995/*1.999*	**1.985/*1.989***	1.979/*1.989*		
	HHHHH	2.225/*2.134*	2.225/*2.131*	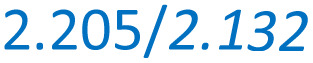		
	LLHLL	1.992/1.996	**2.000/*1.998***	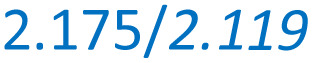		
2	LLLLL	1.985	**1.978**	1.992		
	HHHHH	2.189	2.182	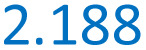		
	HLHLH	2.166	**1.997**	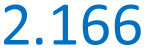		
2a	LLLLL	1.983	**1.989**	1.978		
	HHHHH	2.167	2.180	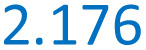		
	HLHLH	2.152	**2.012**	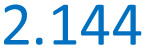		
3	LLLLL	2.001	1.994	1.991	1.986	1.996
	HHHHH	2.189	2.177	2.186	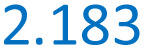	2.182
	LHLHL	2.000	2.159	2.009	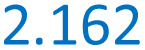	2.008

Before discussing the obtained energies and thermodynamic functions, we briefly review the intramolecular interactions between ligands and anions/solvated water. The relevant interaction patterns are discussed in detail in the SI (Fig. S5–S8). As pointed previously by Vela and Paulsen,^18^ the calculation of several spin isomers for different modifications of the second coordination sphere provides a wealth of structural data that will not be discussed in this paper. Here, we would like to indicate the most important patterns. The first interaction mode is the hydrogen bond between the anion or water and the C–H protons of the triazole ring. Typically, the anion or water oxygen forms a bridge between two such hydrogens of the neighbouring rings. The second one involves the hydrogen bonding between anion/water and 4-substituent of the triazole, *i.e.* either H or NH_2_ group. The inspection of the obtained LS structures of the heptanuclear models reveals 24F⋯H contacts shorter than 3.0 Å for 1. The same distance criterion reveals 42C⋯H contacts for 2 and 25Cl⋯H and 30O⋯H contacts for 2a. The model of 3 reveals 56 contacts between ligand hydrogens and the nitrate oxygens.

This sort of interactions is likely to influence the energy of the spin transitions and affect the elasticity of the 1D chain. Notably, for 1 and 2 with only the F–H and Cl^−^–H interactions the LS → HS transition leads to the increase of the corresponding distances.

On the other hand, there is no clear effect of the spin state on the intermolecular interactions in 2a involving the O(H_2_O)···HC(triazole) OH_2_⋯Cl and NH_2_⋯Cl interactions. For 3, the shortening of nitrate-O contacts with the triazole ring hydrogen is observed on HS to LS switching and the opposite effect occurs for nitrate-*O*-amine protons contacts.

With five inner centres that may be either in HS or LS state there are twenty possible spin isomers:^1^

- Two with all inner Fe-centres being in either LS or HS state, that are further denoted as HHHHH and LLLLL, respectively, corresponding to *x*_HS_ of 1 and 0, respectively.

- Three with one HS centre and four LS ones (the HS defect in LS matrix, corresponding to L^4^H configuration, *x*_HS_ = 0.2), denoted as HLLLL, LHLLL and LLHLL.

- Three with one LS centre and four HS ones (the LS defect in HS matrix, corresponding to LH^4^ configuration, *x*_HS_ = 0.8), denoted as LHHHH, HLHHH and HHLHH.

- Six with three LS and two HS centres (corresponding to L^3^H^2^ configuration, *x*_HS_ = 0.4), further named as “block” (LLLHH), “alternate” (LHLHL), “two pair” (LHHLL), “three” (HLLLH) and two “one pair” (LLHLH and HLLHL).

- Six with three HS and two LS centres (corresponding to L^2^H^3^ configuration, *x*_HS_ = 0.6), again named as “block” (LLHHH), “alternate” (HLHLH), “three” (LHHHL), “two pair” (HLLHH) and two “one pair” (HHLHL and LHHLH).

For each spin isomer the geometry optimisation, followed by normal modes calculation was performed with the B3LYP* functional^71^ and the CEP-31G basis set,^63^ the combination used by us previously to model the 1D SCO chains under study.^47^ The coordinates of all 20 model structures of spin isomers are listed in SI.

The obtained electronic energies calculated relative to those of the LLLLL models are shown in Tables 2, 3, 4 and 5 for 1, 2, 2a and 3, respectively.

**Table 2:** Calculated electronic energies, differences of zero-point vibrational energies and *H*_strain_ (in kJ mol^−1^) parameters of all spin isomers of the heptanuclear model of 1 (*E*_el_ and ZPE assumed to be 0) and the derived values of *H*_coop_ and its analogue for Fe(2). The values of *E*_ad_ calculated and derived from the calorimetric results are given

	*E*_el_				*H*_strain_			B3LYP D3/opt^c^
	B3LYP*		B3LYP^a^	CAM-B3LYP^a^	B3LYP*	B3LYP^a^	CAM-B3LYP^a^	
	*E*_el_	ΔZPE						
LLHLL	55	−12	28	25	13	13	11	18
LHLLL	44	−11	17	15	2	2	1	8
HLLLL	40	−12	13	11.5	−2	−2	−3	4
HLLHL	83.5	−23	29	26.5	0	−1	−2	13
LHHLL	88	−23	35	31	4	5	3	16
LLHLH	94	−23	40	36	10	10	8	10
HLLLH	80	−24	26.5	23	−4	−4	−5	8
LHLHL	86	−22	33	29	2	3	1	16
LLLHH	74	−23	21	18.5	−10	−9	−10	4
HLLHH	114	−35	35	30	−11	−11	−12	8
HLHHL	127	−35	47	41.5	2	1	−1	13
HLHLH	119	−34	39	34	−6	−7	−8	12
LHHHL	121	−34	41	36	−4	−5	−6	13
LHLHH	117	−34	37.5	32.5	−8	−8	−10	11
LLHHH	118	−35	38	34	−7	−8	−8	8
HHLHH	176	−50	69.5	64.5	9	9	8	7
HLHHH	178	−50	73	68	11	12	12	6
LHHHH	180	−50	75	69	13	14	13	7
HHHHH	209	−62	76	70.5				
*E*_ad_	42		15	14				16
*E* ^cal^_ad_		28.5			*E* ^cal^_ad_ = Δ*H*^cal^_HL_ (23.4) − Δ*E*_vib_ (360 K)^d^			
*H*_coop_	22		21.5	19				25
*H*_coop_ Fe(2)^b^	13		14	12.5				

a Calculated for the geometry optimised with B3LYP* using B3LYP or CAM-B3LYP.

b *H*_coop_ calculated for the Fe(2) centre, equal to the difference between *E*_HLLLLLH_ → *E*_HLHLLLH_ and *E*_HHLHHHH_ → *E*_HHHHHH_ spin transition energies.

c Optimised with B3LYP and D3 dispersion correction.

d (*E*_vib_(HHHHH) − *E*_vib_(LLLLL))/5, *i.e.* the change of vibrational energy per 1 centre at the experimentally determined *T*_c_.

**Table 3:** Calculated electronic energy differences, zero-point vibrational energies and *H*_strain_ (in kJ mol^−1^) parameters of all spin isomers of the heptanuclear model of 2 (*E*_el_ and ZPE assumed to be 0) and the derived values of *H*_coop_ and its analogue for Fe(2). The values of *E*_ad_ calculated and derived from the calorimetric results are given

	*E*_el_				*H*_strain_			B3LYP D3/opt^c^
	B3LYP*		B3LYP^a^	CAM-B3LYP^a^	B3LYP*	B3LYP^a^	CAM-B3LYP^a^	
	*E*_el_	ΔZPE						
LLHLL	45.5	10	19	16	2.5	3	3	2
LHLLL	62	13	35	31.5	19	19	18	20
HLLLL	52.5	11	26	26	9.5	10	13	11
HLLHL	114	23	61	59	28	29	32	32
LHHLL	99	22	46	40.5	13	14	14	6
LLHLH	97	21	44	42	11	12	15	12
HLLLH	105	22	52	55	19	20	28	23
LHLHL	123	24	68.5	62.0	37	36.5	35	40
LLLHH	106	24	52	50	20	20	23	20
HLLHH	142	32	61	63	13	13	23	24
HLHHL	147	34	66.5	62	18	18.5	22	13
HLHLH	148	32	69	68	19	21	28	23
LHHHL	151	33	71	63	22	23	23	11
LHLHH	166	35	85	81	37	37	41	39
LLHHH	130	33	50	42	1	2	2	0
HHLHH	189	43	81	75	17	17	21	12
HLHHH	176	42	69	70	4	5	16	16
LHHHH	183	45	74	62	11	10	9	12
HHHHH	215	54	80	67				
*E*_ad_	43		16	13				
*E* ^cal^_ad_		13.5			*E* ^cal^_ad_ = Δ*H*^cal^_HL_ (8) − dΔ*E*_vib_ (340 K)			
*H*_coop_	19.5		20	24				14
*H*_coop_ Fe(2)^b^	23		24	34.5				

a Calculated for the geometry optimised with B3LYP* using B3LYP or CAM-B3LYP.

b *H*_coop_ calculated for the Fe(2) centre, equal to the difference between *E*_HLLLLLH_ → *E*_HLHLLLH_ and *E*_HHLHHHH_ → *E*_HHHHHH_ spin transition energies.

c Optimised with B3LYP and D3 dispersion correction.

d (*E*_vib_(HHHHH) − *E*_vib_(LLLLL))/5, *i.e.* the change of vibrational energy per 1 centre at the experimentally determined *T*_c_.

**Table 4:** Calculated electronic energies, zero-point vibrational energies and *H*_strain_ (in kJ mol^−1^) parameters of all spin isomers of the heptanuclear model of 2a (*E*_HLLLLLH_ assumed to be 0) and the derived values of *H*_coop_ and its analogue for Fe(2). The values of *E*_ad_ calculated and derived from the calorimetric results are given

	*E*_el_				*H*_strain_			B3LYP D3/opt^c^
	B3LYP*		B3LYP^a^	CAM-B3LYP^a^	B3LYP*	B3LYP^a^	CAM-B3LYP^a^	
	*E*_el_	ΔZPE						
LLHLL	70	−14	42	38	18	17	13	17
LHLLL	60	−13	33	32	8	8	7	7
HLLLL	63	−12	36	37	11	11	12	11
HLLHL	122	−25	68	68	18	18	18	18
LHHLL	115	−26	61	57	12	11	7	9
LLHLH	132	−26	76	74	28	26	23	28
HLLLH	125	−24	71	73	21	21	23	22
LHLHL	117	−24	62	63	13	13	13	13
LLLHH	110	−25	56	58	6	6	7	6
HLLHH	172	−37	91	94	17	17	18	16
HLHHL	176	−38	95	92	21	20	17	20
HLHLH	193	−38	111	109	37	36	34	40
LHHHL	170	−35	91	94	15	16	18	2
LHLHH	167	−36	86	89	12	11	13	11
LLHHH	165	−38	84	82	10	9	7	9
HHLHH	218	−48	110	114	10	10	13	9
HLHHH	227	−50	118	117	19	18	17	19
LHHHH	209	−50	101	100	2	1	0	0
HHHHH	260	−62	125	126				
*E*_ad_	52		25	25				34
*E* ^cal^_ad_		21			*E* ^cal^_ad_ = Δ*H*^cal^_HL_ (15.5) − dΔ*E*_vib_ (330 K)			
*H*_coop_	28		21.5	19				25
*H*_coop_ Fe(2)^b^	27		14	12.5				

a Calculated for the geometry optimised with B3LYP* using B3LYP or CAM-B3LYP.

b *H*_coop_ calculated for the Fe(2) centre, equal to the difference between *E*_HLLLLLH_ → *E*_HLHLLLH_ and *E*_HHLHHHH_ → *E*_HHHHHH_ spin transition energies.

c Optimised with B3LYP and D3 dispersion correction.

d (*E*_vib_(HHHHH) − *E*_vib_(LLLLL))/5, *i.e.* the change of vibrational energy per 1 centre at the experimentally determined *T*_c_.

**Table 5:** Calculated electronic energies, zero-point vibrational energies and *H*_strain_ (in kJ mol^−1^) parameters of all spin isomers of the heptanuclear model of 3 (*E*_HLLLLLH_ assumed to be 0) and the derived values of *H*_coop_ and its analogue for Fe(2). The values of *E*_ad_ calculated and derived from the calorimetric results are given

	*E*_el_				*H*_strain_			B3LYP D3/opt^c^
	B3LYP*		B3LYP^a^	CAM-B3LYP^a^	B3LYP*	B3LYP^a^	CAM-B3LYP^a^	
	*E*_el_	ΔZPE						
LLHLL	52	−11	25	25	15	17	17	11
LHLLL	65	−10	37	31	27	22	22	5
HLLLL	41	−11	20	17	4	12	9	−7
HLLHL	93	−21	39	33	19	16	16	9
LHHLL	115	−22	61	55	40	38	38	5
LLHLH	85	−20	33	33	11	16	16	2
HLLLH	81	−22	27	25	6	8	8	−10
LHLHL	118	−18	64	55	44	38	38	25
LLLHH	84	−21	30	25	10	8	8	4
HLLHH	116	−31	35	30	5	4	4	18
HLHHL	131	−31	53	52	20	26	26	10
HLHLH	117	−30	39	35	6	10	10	−3
LHHHL	148	−31	67	58	36	32	32	10
LHLHH	130	28	48	42	18	17	17	35
LLHHH	139	−32	42	42	10	16	16	20
HHLHH	166	−42	58	49	18	15	15	8
HLHHH	161	−41	55	46	13	12	12	−10
LHHHH	172	−44	64	52	24	18	18	−2
HHHHH	185	−52	52	43				
*E*_ad_	37		8.5	10				18
*E* ^cal^_ad_		27			*E* ^cal^_ad_ = Δ*H*^cal^_HL_ (23.1) − dΔ*E*_vib_ (330 K)			
*H*_coop_	33		31	31				33
*H*_coop_ Fe(2)^b^	41		39	34				

a Calculated for the geometry optimised with B3LYP* using B3LYP or CAM-B3LYP.

b *H*_coop_ calculated for the Fe(2) centre, equal to the difference between *E*_HLLLLLH_ → *E*_HLHLLLH_ and *E*_HHLHHHH_ → *E*_HHHHHH_ spin transition energies.

c Optimised with B3LYP and D3 dispersion correction.

d (*E*_vib_(HHHHH) − *E*_vib_(LLLLL))/5, *i.e.* the change of vibrational energy per 1 centre at the experimentally determined *T*_c_.

The comparison of the so defined relative energies of the spin isomers allows the following conclusions (see Tables 2–5 and Fig. S1–S4, for the *E*_el_ values):

(i) The electronic energy increases with the number of HS centres in the inner five-nuclear Fe-fragment of the heptanuclear model.

(ii) Each spin isomer containing the same ratio of HS to LS centres reveals a different electronic energy. There is hardly a pattern of stabilisation/destabilisation of a given spin isomer that is common for all systems. For example, while both “block” spin isomers LLHHH and HHHLL are of lowest energy of all L^2^H^3^ isomers (for 1, 2a and 3, respectively, see Tables 2, 4 and 5), this is not the case for 2 (*cf.*Table 3). The “block” LLLHH isomers are of the lowest energy for 2 and 2a but not for 1 and 3. Similarly, the “alternate” antiferroelastic LHLHL is the most destabilised among L^3^H^2^ of 1 and 3 (Tables 2 and 5), but not for 2 and 2a (Tables 3 and 4).

(iii) The largest energy gap occurs between the LLLLL isomer and the one with one HS defect (LLHLL, LHLLL and HLLLL). The minimal values are 40, 45.5, 60 and 41 kJ mol^−1^ for 1, 2, 2a and 3, respectively. This implies a high stabilisation of the LS state in the pure LS matrix. Still, there is no pattern of a particular stabilisation or destabilisation of the particular spin isomer with one HS defect. The centrosymmetric LLHLL isomer is of lowest energy for 1 and 3 (see Tables 2 and 5) while it is of highest energy for 2 and 2a (see Tables 3 and 4).

In this respect complex 2 is particular, revealing a significant energy gap between the L^2^H^3^ and LH^4^ spin isomers. The smallest energy gap is that between the LHLHH and LLHHH ones (36 kJ mol^−1^).

(iv) The calculated electronic energies of LLLLL → HHHHH transitions per one switching centre are 42, 43, 52 and 37 kJ mol^−1^ for 1, 2, 2a and 3, respectively. The calorimetrically obtained values (see Table 7) are respectively 23.4, 9, 12 and 23 kJ mol^−1^. The zero-point corrected electronic energies per one center (Tables 2–5) are 29.4, 32.2, 39.6 and 26.6 kJ mol^−1^. Thus, the calculated LLLLL → HHHHH transition energies for the 1D models of the system under study do not fit to the experimentally observed trend of the spin transition energies increasing in the 3 < 1 < 2 < 2a sequence.

**Table 6:** Calculated electronic energies, and *H*_strain_ (in kJ mol^−1^) parameters of all spin isomers of heptanuclear model of 1 (*E*_HLLLLLH_ assumed to be 0) and the derived values of obtained with B3LYP and CAM-B3LYP functionals with and without dispersion correction, and with PBEh functional

	B3LYP/D3		B3LYP		CAM-B3LYP/D3		CAM-B3LYP		PBEh	
	*E*_el_	*H*_strain_	*E*_el_	*H*_strain_	*E*_el_	*H*_strain_	*E*_el_	*H*_strain_	*E*_el_	*H*_strain_
LLHLL	34	18	28	19	29	18	26	18	−2	19
LHLLL	24	8	18	8	20	8	15	7	−10	8
HLLLL	20	4	14	4	16	4	12	4	−17	4
HLLHL	44	13	31	12	35	12	28	11	−33	9
LHHLL	47	16	36	17	39	16	32	16	−26	16
LLHLH	42	10	29	10	33	8	26	9	−31	11
HLLLH	40	8	27	8	31	8	24	8	−33	9
LHLHL	47	16	34	15	38	15	30	14	−28	14
LLLHH	35	4	22	3	27	4	19	3	−39	3
HLLHH	56	8	36	8	43	7	32	7	−55	8
HLHHL	61	13	42	14	48	13	37	13	−49	14
HLHLH	60	12	41	12	46	11	35	11	−51	12
LHHHL	61	13	43	14	48	13	37	13	−50	13
LHLHH	59	11	39	10	46	11	34	9	−53	10
LLHHH	55	8	36	8	43	8	32	7	−51	12
HHLHH	70	7	44	6	53	6	38	5	−78	6
HLHHH	70	6	45	6	53	6	38	6	−77	7
LHHHH	71	7	46	8	54	7	40	7	−76	8
HHHHH	79		48		59		41		−105	
*E*_ad_	16		10		12		8		−21	
*H*_coop_	25		24		24		23		25	

**Table 7:** Phase transition temperatures and thermodynamic parameters as deduced from DSC measurements and calculated (B3LYP*) vibrational entropies

1D chain	#^b^	*T*_max_^↑^ [K]	*T*_max_^↓^ [K]	Δ*T* [K]	ss%^a^	Δ*H*_HL_ [kJ mol^−1^]	Δ*S*_HL_ [J mol^−1^ K^−1^]	Δ*S*_vib_ (DFT)
[Fe(Htrz)_2_trz]BF_4_ (1)	1^c^	387	343	44	86	23.4	64.1	50.7
								73 (390 K)
								72 (340 K)
	2^c^	390	347	43	86	23.4	63.5	50.2
	3^c^	391	344	47	86	24	65.3	52
[Fe(NH_2_trz)_3_]Cl_2_ (2)	1^c^	343	335	8	f	8.93	26.06	12.73
								52 (310 K)
[Fe(NH_2_trz)_3_]Cl_2_·2.5H_2_O (2a)	1^c^	332	311	21	f	12.22	39.38	23.05
								60 (330 K)
[Fe(NH_2_trz)_3_](NO_3_)_2_ (3)	1^d^	347	314	33	92	23(1)	69.6(1)	56.2
								70 (350 K)
								56 (310 K)
[Fe(NH_2_trz)_3_]TiF_6_·0.5H_2_O	1^e^	210	200	10	72	7.1	34.5	21.1
[Fe(NH_2_trz)_3_]TiF_6_·H_2_O	1^e^	206	201	5	75	7.3	36	22.6
[Fe(NH_2_trz)_3_]ZrF_6_·0.5H_2_O	1^e^	233	209	24	85	7.4	33.6	20.2
[Fe(NH_2_trz)_3_]ZrF_6_	1^e^	256	220	36	85	6.7	30.5	17.1
[Fe(NH_2_trz)_3_]SnF_6_·0.5H_2_O	1^e^	224	203	21	75	6.3	31.2	17.8
[Fe(NH_2_trz)_3_]SnF_6_·H_2_O	1^e^	244	224	20	80	8	34.3	20.9
[Fe(NH_2_trz)_3_]TaF_7_·3H_2_O	1^e^	206	197	9	72	5.9	30	17
[Fe(NH_2_trz)_3_]GeF_6_·H_2_O	1^e^	213	211	2	62	6.4	30	17
[Fe(NH_2_trz)_3_]GeF_6_·0.5H_2_O	1^e^	213	207	6	58	6.9	32	19

a ss: switching sites determined by ^57^Fe Mössbauer spectroscopy when comparing the highest and lowest temperature spectra.

b Run numbering.

c This work.

d Ref. 69.

e Ref. 64*a*.

f 100% spin conversion assumed on the basis of magnetic and NIS data (see Experimental section for discussion).

(v) Finally, it is of interest how much the electronic energy of the spin isomers containing the same number of HS and LS centres depends on the distribution of the centres of a given spin. For example, what is the difference between L^4^H spin isomer LLHLL, LHLLL and HLLLH. The inspection of the Tables 2–5 reveals that the largest difference of 37 kJ mol^−1^ is obtained for LHLHL and HLLLH spin isomers of 3. For the L^2^H^3^ isomers of 2, the energy difference between LHLHH and LLHHH is 36 kJ mol^−1^. These values are derived for the systems of the same multiplicity and are supposedly independent from the applied functional (*vide infra*). Consequently, one may state that the energy differences between the different pattern of spin distribution for a given number of HS and LS centres are comparable or larger than the typical spin transition energies that span the range of 2–20 kJ mol^−1^ as observed for 1D triazole-based complexes.^64^ Note that zero-point vibrational energies effectively do not depend on the spin distribution in spin isomers containing the same number of HS and LS centres (see Tables 2–5). Moreover, with the increasing number of the HS centres the ZPE decreases linearly with the number of the HS centres. For example, for 1 the ZPE is lower at −11/-12 kJ mol^−1^ for L^4^H isomers compared to the fully LS model. The corresponding values for L^3^H^2^, L^2^H^3^, LH^4^ and full HS spin isomers are respectively −23, −34 to −35, −50 and −62 kJ mol^−1^.

In the next step, the cooperativity parameter *H*_coop_ was derived for the central Fe(1) and for Fe(2) (Scheme 1). As defined in ref. 50, *H*_coop_ corresponds to the difference of calculated spin transition energies within the matrix of LS and HS neighbours. Hence, *H*_coop_ for Fe(1) is equal to [(*E*(LLHLL) − *E*(LLLLL)) − (*E*(HHHHH) − *E*(HHLHH))], while *H*_coop_ for Fe(2) is equal to [(*E*(LHLLL) − *E*(LLLLL)) − (*E*(HHHHH) − *E*(HLHHH))]. *H*_coop_ for Fe(1) results to 22, 19.5, 28 and 33 kJ mol^−1^ for 1, 2, 2a and 3 respectively. For Fe(2) the corresponding values are 13, 23, 27 and 41 kJ mol^−1^ (Tables 2–5). As stated above (ref. 50) these values reflect the amount of the deformation of the FeN_6_-core by the matrix of a different spin for the LS and HS centres. Another parameter is the stress due to neighbourhood of both LS and HS centre, being the net result of deformation (strain) of all centres from their regular HS and LS geometry. To derive it, one needs to assume that the calculated electronic energy *E*_el_ of a given spin isomer relative to the LLLLL one is the sum of two contributions: (i) the electronic energy (dependent on the exchange–correlation functional) of a single-centre spin transition (*E*_ad_). The latter is independent on the position of the centre in the chain. (ii) The stress due to geometric strain in a given spin isomer. Hence, the relative electronic energy of a given isomer containing *n*HS centres and 5-*n*LS centres is:

1 *E*_el_(H_*n*_L_5−*n*_) = *nE*_ad_ + *H*_strain_

where *H*_strain_ is the cumulative stress due to the deformation of all HS and all LS centres from their geometries in the pure HS and LS phases, respectively. It becomes zero for the pure LS and HS phases.

To derive *H*_strain_ for our models, we estimated *E*_ad_ to be 1/5 of the energy difference between LLLLL and HHHHH spin isomers, assuming a negligible stress in the heptanuclear model molecule for the LS and the HS state (*i.e.* for the LLLLL and HHHHH systems). We obtain 42, 43, 52 and 37 kJ mol^−1^ for 1, 2, 2a and 3 respectively. With these numbers we derive *H*_strain_ for all spin isomers that are listed in Tables 2–5. The NH_2_trz complexes 2, 2a and 3 display a similar pattern of *H*_strain_ being the largest for the L^3^H^2^ and L^2^H^3^ spin isomers with maximal values of +44 kJ mol^−1^ for the “alternate” LHLHL spin isomer of 3. The other spin isomers L^3^H^2^ and L^2^H^3^ display a very low *H*_strain_ with that for the “block” LLHHH isomer of 2 being only 1 kJ mol^−1^. On the other hand, the *H*_strain_ values obtained for 1 reveal an opposite trend. Particularly for L^2^H^3^ and L^3^H^2^ spin isomers *H*_strain_ is negative, indicating that strain induces a spin stabilizing effect. The structural reason for this effect is not immediately clear, yet with B3LYP/D3 optimisation only the positive values of *H*_strain_ are obtained (see Table 2).

It is of interest whether the values of *H*_strain_ are reproducible using other functionals since any functional chosen leads to different absolute electronic energy values.^15^ Therefore, for all structures optimised with B3LYP*, the calculations of the electronic energies were performed also with CAM-B3LYP^65^ and B3LYP^66^ using the CEP-31G basis set (Tables 2–5). The calculated values of *E*_ad_ decrease from 42–43 kJ mol^−1^ to 16–13 kJ mol^−1^ on changing from B3LYP* to B3LYP/CAM-B3LYP for 1 and 2, from 52 to 25 kJ mol^−1^ for 2a and from 37 to 10 and 8.5 kJ mol^−1^, respectively for 3. Nevertheless, the energy sequence as well as *H*_coop_ and *H*_strain_ are comparable for all three functionals. There are some exceptions for 2 calculated with CAM-B3LYP, in line with previous findings^51^ but *H*_coop_ and *H*_strain_ of 2 are equivalent. Assuming a constant value of *E*_ad_ the cooperativity parameter *H*_coop_ can by expressed for the central Fe(1) as *H*_coop_ (Fe(1)) = *H*_strain_(LLHLL) − *H*_strain_(HHLHH). Importantly, for the LLLLL → LLHLL transition the strain is introduced, while for the HHLHH → HHHHH one it is released. Noteworthy, the energy of elastic amplification observed after photo-induced LS → HS transition^67^ can be also expressed in term of *H*_coop_ and *H*_strain_.^2^

Based on the electronic energies of spin isomers collected in Tables 2–5 several other parameters characterizing the short-range interactions may be derived (see Table S1). These results are presented in SI. By and large, the following conclusions may be drawn:

(i) The presence of HS neighbours generally favours the LS to HS transition four all four modelled systems.

(ii) This effect weakens with the distances between the HS centre and the switching centre only when the HS centre can be considered as a defect in a LS matrix. For example, the LLLLL to HLLLL and HLLLL to HLLLH transition display practically no difference for all modelled molecules, while the LHHHL to LHHHH and LHHHH to HHHHH transition energies show large differences (*e.g.* from −12 to 30 kJ mol^−1^ for 2a and 1, respectively, 29 and 41, and 59 and 29 kJ mol^−1^). The exception is 2, for which these two transitions reveal the same energies. This suggests that the elastic effect of the defect has a higher range in the more elastic matrix of the HS centres. This shows that the short-range interactions affect centres at a distance of *ca.* 1.6 nm in the HS matrix. On the other hand, there is no difference in the calculated spin transition energies of LLLL**L** → LLLL**H** and **L**LLLH → **H**LLLH transitions.

(iii) The obtained parameters (see Table S1) for 2 are somewhat lower than for the other systems while 3 is exceptional in showing practically no zero values. Considering that NH_2_trz–Cl (with no additional ligand–water interaction) contacts are supposed to be the weakest while those of NO_3_–NH_2_trz type are supposed to be the strongest, we conclude that the strong anion–ligand interactions may increase the transfer of the elastic distortion.

In next step, we investigated how *H*_strain_ differs if the optimisations and energy calculations are performed with other functionals. In this case we carried out the modelling with B3LYP functional including dispersion correction. The obtained values of *H*_strain_ and *H*_coop_ are shown in Tables 2–5 the calculated electronic energies for 1 given in Table 6, while those for 2, 2a and 3 are given in, together with the ZPE corrections SI (Tables S3–S5). The values of ZPE corrections for 1 obtained with different functionals are collected in Table S6.

The comparison of *H*_strain_ obtained for the structures optimised with B3LYP* and B3LYP/D3 reveals a surprising pattern (see Tables 2–5). Nearly the same values were derived for 2a (with exception of the LHHHL isomer). Fairly similar values were found for 2, the most significant differences found for LHHHL (11 and 22 kJ mol^−1^ for B3LYP/D3 and B3LYP*, respectively) and HLHHH (16 and 4 kJ mol^−1^, respectively). Noteworthy, the latter two values obtained with B3LYP/D3 are very close to those obtained with CAM-B3LYP for the geometry obtained with B3LYP* (see Table 3). On the other hand, the *H*_strain_ parameters derived with B3LYP/D3 for 1, show a different pattern than those derived with B3LYP*, particularly for the L^3^H^2^ and L^2^H^3^ spin isomers where the negative values obtained with B3LYP* turn positive for B3LYP/D3 (see Table 2). For 3 this discrepancy is also observed, again with three values of *H*_strain_ revealing the negative values for B3LYP/D3 (see Table 5). On the other hand, the values of *H*_coop_ seem to be closer for the two functionals yielding 22 and 25 kJ mol^−1^ for B3LYP* and B3LYP/D3 for 1, and respectively 19.5 and 14 kJ mol^−1^ for 2, 28 and 25 kJ mol^−1^ for 2a and 33 kJ mol^−1^ for both functionals for 3. That means that the *H*_strain_ parameters for LLHLL and HHLHH are less dependent on the choice of the functional.

To get a further insight into this dependence we performed optimisations with B3LYP and CAM-B3LYP with and without dispersion correction and additionally for PBEh functional for 1 as an example of another hybrid functional, not related to B3LYP. The results are given in Table 6. Additionally, the CAM-B3LYP/D3 and PBEh modelling was done for 2, the results are shown in Table S3. The results show that within these functionals quite consistent values of *H*_strain_ are obtained. The largest differences of *H*_strain_ are obtained for the LLHHH spin isomer, with all B3LYP and CAM-B3LYP (with or without dispersion) functionals yielding the values of 7–8 kJ mol^−1^ while PBEh yielded 12 kJ mol^−1^. The differences between results with and without dispersion correction are negligible. Again, the obtained *H*_coop_ values lie all in the range 23–25 kJ mol^−1^. Further calculations of *H*_coop_ with other functionals (TPSS and TPSSh) show quite constant values for each modelled system (see Table S2). The data obtained for 2 with B3LYP/D3, CAM-B3LYP/D3 and PBEh are also quite close (see Table S3), the exceptions being “two pair” LHHLL and HLLHH spin isomers (*H*_strain_ for LHHLL equal to 6, 4 and 13 kJ mol^−1^) for B3LYP/D3, CAM-B3LYP/D3 and PBEh, respectively, the corresponding values for HLLHH being 24, 5 and 3 kJ mol^−1^. To summarize, the values of *H*_strain_ for different spin isomers are comparable if for the same geometry (obtained with B3LYP*) the energies are calculated with B3LYP and CAM-B3LYP. Whether that obtained with B3LYP* (optimisation and energy calculations) differ from that obtained for the optimisation and energy calculations with B3LYP, CAM-B3LYP or PBEh depends on the model molecule. Within the last series of functionals they match well. The reason for the different values obtained with B3LYP* is not immediately clear, yet the comparison of the selected Fe–N distances suggests that the obtained values of *H*_strain_ correlate with the ability of different functionals to give the HS Fe–N bonds lengths that differ less than 0.01 Å (see Table S7).

### Thermodynamics of spin transition

The temperature dependencies of the Gibbs free energy changes for the LLLLL → L_5−*n*_H_*n*_ spin transitions have been computed for the 19 spin isomers. Our calculations are based on the approach described in ref. 42, *i.e.* according to the following formula:

2 Δ*G*(*T*) = Δ*E*_el_ + Δ*H*_vib_(*T*) − *T* (Δ*S*_vib_(*T*) + Δ*S*_mag_ + Δ*S*_mix_ + Δ*S*_conf_)

where *E*_el_ denotes the calculated electronic energies, *n* the number of HS centres in the inner chain of the five irons, Δ*S*_mag_ = *R*[ln(2*S* + 1)_LS_^*n*^_HS_^(5−*n*)^/ln(2*S* + 1)_LS_^5^] (ref. 1*a*). The latter is the entropy change due to change of multiplicity on going from the pure LS state to a given spin isomer L^*n*^HS^5−*n*^. In simple form, it is equal to *nR* ln 5. Δ*S*_mix_ is the mixing entropy change given by the formula: Δ*S*_mix_ = −*R*(*x*_HS_ ln *x*_HS_ + (1 − *x*_H_)ln(1 − *x*_HS_)). Note that *x*_HS_ in our case is 0.2 for L^4^H, 0.4 for L^3^H^2^, 0.6 for L^2^H^3^ and 0.8 for LH^4^ spin isomers. Δ*S*_conf_ reflects the probability of formation of a given isomer. It is zero for the centrosymmetric isomers and *R* ln 2 for those bearing no inversion centre. It may be given by the formula Δ*S*_conf_ = *R* ln(*nk*), were *nk* denotes the number of molecular permutations corresponding to a given spin isomer. For example, there are two ways the isomer HLLLL could be formed, but only one for LLHLL one. *E*_vib_(*T*) is the vibronic energy and Δ*S*_vib_(*T*) denotes the change of vibronic entropy upon the spin transition. Both, *E*_vib_(*T*) and Δ*S*_vib_(*T*) have been obtained by DFT based normal mode calculations.

Additionally, the Δ*G*(*T*) dependencies were also obtained using the *E*_el_ values calculated with B3LYP and CAM-B3LYP and all other parameters obtained with B3LYP* (see further discussion). For these calculations the geometries optimised with B3LYP* were used for the point-energy calculations of energies (followed by the stability check, *vide infra*). In this way the only parameter that is changed is the electronic energy characteristic for a given functional.

**Fig. 1:**
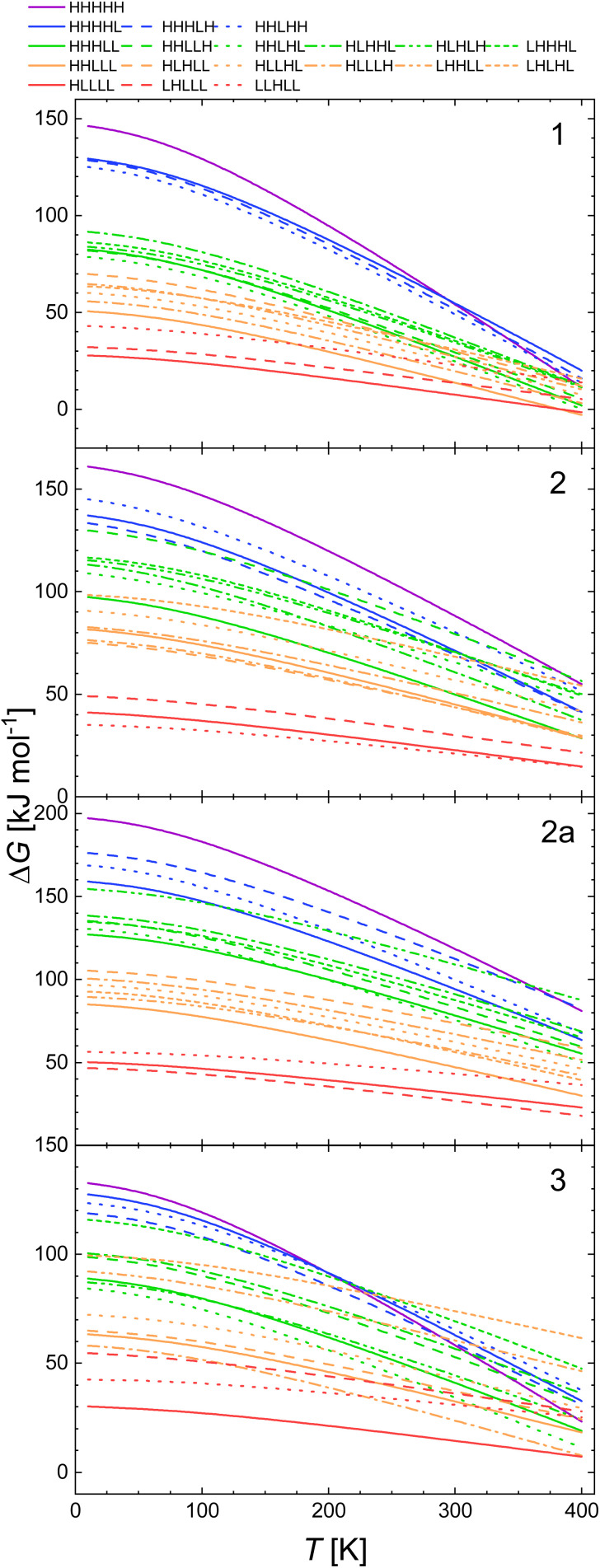
Calculated temperature dependence of Δ*G* of the spin transition from the LLLLL spin isomers to all spin isomers of heptanuclear models of 1, 2, 2a and 3 obtained with B3LYP* functional and CEP-31G basis set.

**Fig. 2:**
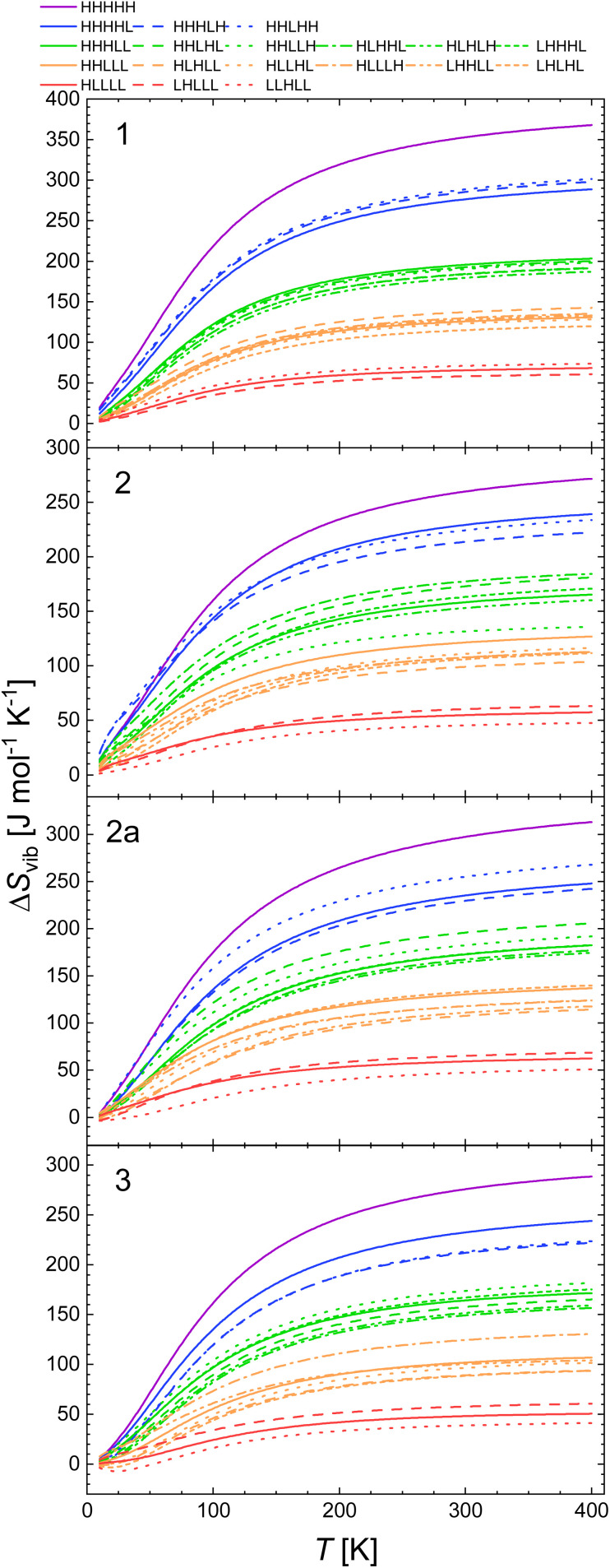
Calculated temperature dependence of Δ*S*_vib_ of the spin transition from the LLLLL spin isomers to all spin isomers of heptanuclear model of 1, 2, 2a and 3 obtained with B3LYP* functional.

The plots of Δ*G*(*T*) calculated with the B3LYP* functional of all spin isomers of 1, 2, 2a, and 3 are shown in Fig. 1. The respective calculated plots of vibrational entropy obtained for B3LYP* functional are shown in Fig. 2.

There are distinct values of Gibbs free energy and vibration entropy for each spin isomer of a given multiplicity. This means that electronic and vibrational energy, Gibbs free energy and entropy are dependent on the distribution of the LS and HS centres within the pentanuclear fragment of the chain. Accordingly, the different distribution leads to different level of strain extorted by the proximity of the neighbours of different spin. The so obtained Δ*G*(*T*) curves show that the combination of strain and entropic effects leads to a situation where the thermodynamic stability of a given isomer is not linearly dependent on the number of HS centres.

This is particularly pronounced for 2, where the LLHHL and LHLHL isomers are up to 15 kJ mol^−1^ less stable than the LLHHH and HHLLH isomers below 100 K (Fig. 2).

According to Fig. 2, the entropy changes with respect to the LLLLL isomer scales with the number of HS centres. Somewhat less obvious, however, is the observation that the higher the multiplicity, the stronger is the dependence of the vibrational entropy on temperature. While for all systems, particularly for 1, Δ*S*_vib_ for model molecules with three HS centres reaches (blue lines in Fig. 4) its maximal value at 250–300 K. For the systems with more than three HS centres Δ*S*_vib_ still grows at higher temperatures: The calculated value of Δ*S*_vib_ for the HHHHH isomer of 2 grows at *ca.* 20 J K^−1^ mol^−1^ between 300 and 400 K. For 1, the vibrational entropy curves for spin isomers containing the same number of HS and LS centres are very close to each other. Those for 2, 2a and 3 deviate more.

### Electronic energy tuning. Δ*G*(*T*) calculated with B3LYP and CAM-B3LYP for the geometries and entropy obtained with B3LYP

Actually, the obtained temperature dependencies of Δ*G*, calculated with the B3LYP* functional do not reveal the properties of the model systems, namely the abrupt spin transition, *i.e.* switching between fully HS and fully LS chains. Instead, differences of the Gibbs free energy values of all possible spin isomers tend to decrease with temperature. The fully LS system is predicted to have the lowest Gibbs free energy up to 400 K. This effect is likely to be due to the propensity of the B3LYP* functional to both lower the energy of the low-spin state and higher for the HS one, thus increasing the spin gap Δ*E*_HL_ = *E*_HS_ − *E*_LS_ (*E*_el_ in notation used here).^15^ Generally, the computed electronic energies of spin transition are highly dependent on correlation-exchange functionals.^14,15^

On the other hand, the differences seem not to be that large in what concerns the vibrational properties and hence the vibrational energy and entropy. Note the importance of the accuracy of calculating Δ*S*_vib_.^68^ For the critical temperature Δ*S*_vib_ = 33 ± 13 J K^−1^ mol^−1^ was obtained for [Fe(phen)_2_(NCS)_2_] using different basis sets. This issue is discussed in detail in SI where we present the results of vibrational entropy calculation obtained with the B3LYP/D3 optimised structures of all spin isomers of 1–3. For 1, the results used with CAM-B3LYP/D3 and CAM-B3LYP and B3LYP with no dispersion correction are given. For 2 the results with CAM-B3LYP/D3 are also given. All calculated harmonic frequencies are given together with *xyz* coordinates in SI.

Thus, we first performed the calculations of the Δ*G*(*T*) dependencies using the vibrational entropy and energy values obtained with B3LYP*, while the electronic energies were computed with the B3LYP functional. The latter is known to give lower energies for the HS isomers of Fe(ii). With CAM-B3LYP a further fine-tuning of the calculated electronic energy is provided. In each case, the geometries obtained with B3LYP* were applied. In the next step, Δ*G*(*T*) curves are given for the models optimised with B3LYP/D3.

We have shown above that the differences of the electronic energies within the series of spin isomers of the same multiplicity, that reflect the strain effects leading to intramolecular cooperativity are overall quantitatively retained, independently on the applied functional based on the B3LYP one. Yet, the relative stability of the isomers containing a given number of HS centres differs according to the relation CAM-B3LYP ≥ B3LYP > B3LYP*. This gives rise to a higher weight of entropic effects in the predicted values of Δ*G*.

**Fig. 3:**
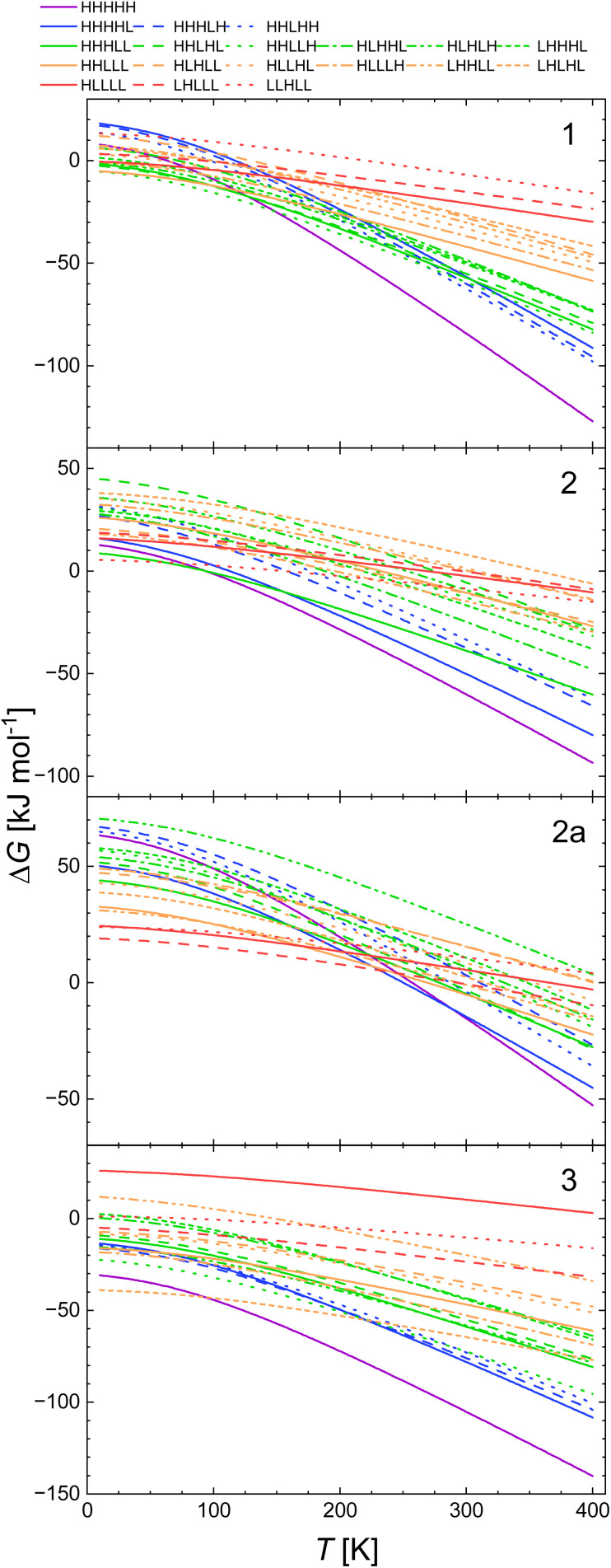
Calculated temperature dependence of Δ*G* of the spin transition from the LLLLL spin isomers to all spin isomers of heptanuclear model of 1, 2, 2a and 3 obtained with CAM-B3LYP functional, using the geometries and vibrational entropies and energies obtained for the B3LYP* functional.

**Fig. 4:**
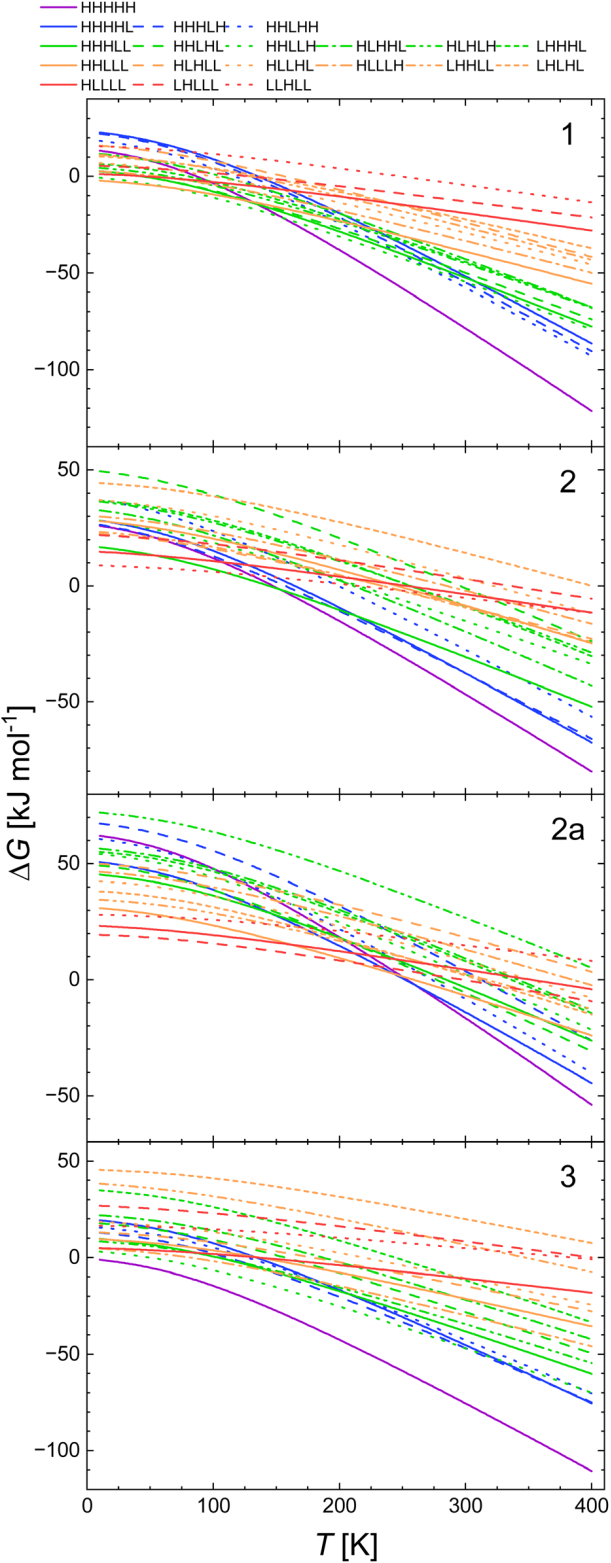
Calculated temperature dependence of Δ*G* of the spin transition from the LLLLL spin isomers to all spin isomers of heptanuclear model of 1, 2, 2a and 3 obtained with B3LYP functional, using the geometries and vibrational entropies and energies obtained for the B3LYP* functional.

The plots of Δ*G*(*T*) calculated with B3LYP and CAM-B3LYP of all spin isomers of 1, 2, 2a and 3 are shown in Fig. 3 and 4.

The inspection of respective figures allows the following conclusions: (i) with the lower value of *E*_ad_ the entropic effects may lead either to (i) a predicted change from the LLLLL ground state to the HHHHH spin isomer for 2, (ii) to HHHHH being the ground state in case of 3, (iii) a predicted change from the LLLLL ground state to the HHHLL isomer for 2a, and (iv) switching between HHHLL and HHHHH isomers in case of 1. For both 1 and 2, both the B3LYP and the CAM-B3LYP functionals predict the purely HS state as the ground state at temperatures above 150–200 K. Fig. 3 and 4 show that the entropy lowers the Gibbs free energy of the pure HS phase with increasing temperature. For 1, for which the strain could have negative values (Table 2) the same effect is obtained while Δ*S*_vib_ is significantly larger than for other complexes under study (Fig. 2).

(ii) With decreasing *E*_ad_ the strain effects may lead to a higher stabilisation of spin isomers having a majority number of HS centres. This is well seen for 2 for which the L^2^H^3^ spin isomers are predicted to be more stable than the L^3^H^2^ ones when *E*_ad_ is calculated with B3LYP and CAM-B3LYP functionals. For 3, this is seen even with B3LYP*. In all cases this effect occurs at low temperatures at which the entropic effects are small.

**Fig. 5:**
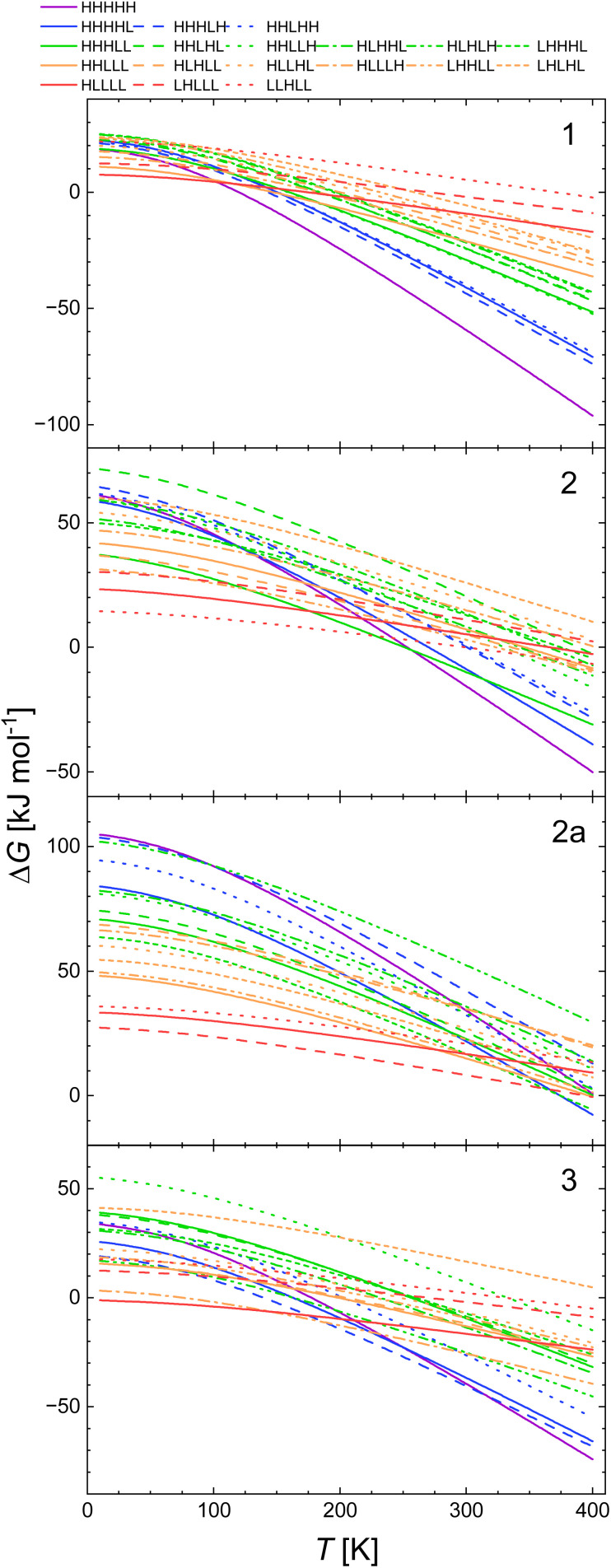
Calculated temperature dependence of Δ*G* of the spin transition from the LLLLL spin isomers to all spin isomers of heptanuclear model of 1, 2, 2a and 3 obtained with B3LYP/D3 functional.

In the next step, we calculated the Δ*G*(*T*) dependencies for all four systems under study using the previously mentioned calculations using B3LYP functional with the dispersion correction D3. Thus, we can compare the results obtained with B3LYP* with those obtained for the B3LYP functional giving another values of *E*_ad_ with the vibronic properties calculated also with B3LYP. The calculated Δ*G*(*T*) curves for the model molecules optimised with this method are given in Fig. 5.

The comparison of the results shown in Fig. 5 with those in Fig. 4, *i.e.* these obtained with B3LYP/D3 with those calculated with B3LYP* for the geometry obtained with B3LYP* reveals that the Δ*G*(*T*) curves for 1 and 2 seems similar with some shift of the temperature at which the HHHHH turns to be ground state to higher temperature for B3LYP/D3. For 2a and 3 the differences are more pronounced. This effect may be related to the different values of *E*_ad_ within the two approaches. The data in Tables 2–5 show that *E*_ad_ are quite close for 1 (15 and 16 kJ mol^−1^ for B3LYP* and B3LYP/D3 geometries, respectively) and for 2 (16 and 17 kJ mol^−1^, respectively). For 2a and 3 they are respectively 25 and 34 kJ mol^−1^, and 8.5 and 18 kJ mol^−1^. The similar pattern was obtained for the optimisation with B3LYP with no dispersion correction and CAM-B3LYP (with and without dispersion correction) for 1 and for CAM-B3LYP/D3 (see SI). In summary, our study shows that for the two modelled systems (1 and 2) *E*_ad_ values obtained with B3LYP and CAM-B3LYP functionals (and geometries from B3LYP*) all HS state turns to be the ground state above 200 K. The B3LYP/D3 approach predicts all HS state to be the ground state of 1 above 100 K and over 200 K for 2. With the B3LYP* geometry, for 2 the pure LS state turns to be the ground state up to 100 K with both CAM-B3LYP and B3LYP obtained energies for 3, the HS ground state is predicted in the whole temperature range. The B3LYP/D3 calculations for 3 predict the all HS state over 300 K.

Thus, our approach suggests that the short-range intra-chain interactions lead to a ferroelastic character of the 1D SCO chains investigated in this study. An experimental determination of *E*_ad_ is possible with calorimetry. Therefore, we decided to determine experimentally SCO transition enthalpies in order to model the SCO behaviour with model molecules of 1–3.

### Calorimetric data as input for DFT modelling

The temperature dependence of the heat capacity, *C*_p_, of 1, 2 and 2a was recorded by DSC. (see chapter Thermal analysis and Experimental and computational methods for details) to derive phase transition temperatures on warming and cooling, hysteresis width, both energy and entropy associated to the spin transition, Δ*H*_HL_ and Δ*S*_HL_ as well as Δ*S*_vib_ (Table 7). Reported data fall in the range of previously reported [Fe(NH_2_trz)_3_]X_2_ complexes with different anions, including 3 (Table 7).

The obtained Δ*H*_HL_ values for 1, 2 and 2a and for 3 (ref. 69) provide the sum of the electronic energy (*i.e. E*_ad_), vibrational energy and the intermolecular energy of the spin transition. We assume that for each spin isomer Δ*G* regarding the LLLLL state per one Fe centre is given by:

3 Δ*G*(*T*) = *n*(Δ*H*_HL_ − Δ*H*_vib_(*T*_c_)/5) + Δ*H*_vib_(*T*) + *H*_strain_ − *T*(Δ*S*_vib_(*T*) + Δ*S*_mag_ + Δ*S*_mix_ + Δ*S*_conf_)

where *n* is the number of the HS centres within the pentanuclear chain. Δ*S*_vib_ and *H*_strain_ denote the vibrational entropy and interaction parameters, respectively (the latter listed in Table 2) obtained with B3LYP*. Δ*H*_HL_ is the calorimetrically determined energy of the all-LS to the all-HS transition. Note that on a contrary to eqn (2) the first term of eqn (3) corresponds to the sum of the electronic and vibronic energies. The former is independent of temperature. An additional contribution to Δ*G* comes from stress for those spin isomers containing the centres of both spin states. Thus, *E*_ad_ can be determined by summing up calorimetrical Δ*H*_HL_ and DFT calculated Δ*H*_vib_(*T*_c_).

**Fig. 6:**
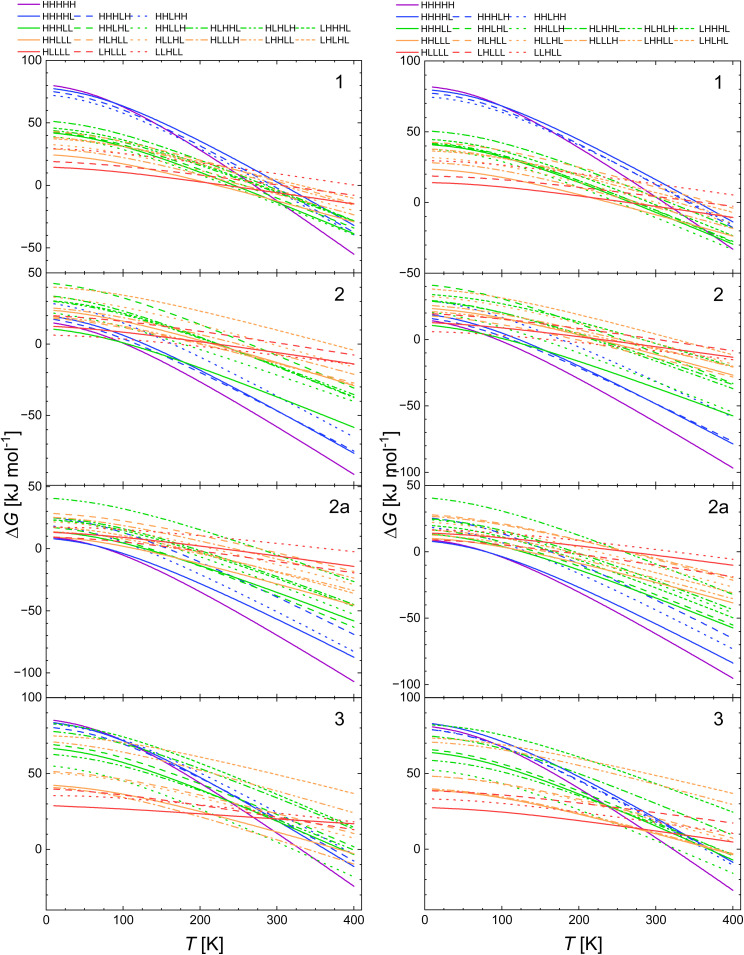
Calculated temperature dependence of Δ*G* of the spin transition from the LLLLL spin isomers to all spin isomers of heptanuclear model of 1, 2, 2a and 3 obtained with the *E*_ad_ value estimated from the calorimetric data and *H*_strain_ for each spin isomer (see eqn (3)), using the geometries, *H*_strain_ values and vibrational entropies and energies obtained for the B3LYP* and B3LYP/D3.

For the LS → HS transition Δ*H*_vib_ is negative as the energy of the stretching vibrations is lowered upon this transition. Therefore, *E*_ad_ is larger than Δ*H*_HL_. The so estimated values of *E*_ad_ are listed in Tables 2–5. For all systems our estimated *E*_ad_ values are significantly lower than the ones calculated with B3LYP*, while for the B3LYP/D3 optimised structures the relation between these values varies between the systems. Thus, for 2 the calorimetrically and B3LYP/D3 calculated values of *E*_ad_ are close to each other (13.5 *vs.* 16 and 17 kJ mol^−1^). For 1, and 3, the values derived from calorimetric data are 9–15.5 kJ mol^−1^ higher than those calculated with the above two functionals. For 2a, on the other hand the calculated *E*_ad_ of 34 kJ mol^−1^ is higher than that determined calorimetrically (21 kJ mol^−1^). The so derived Δ*G*(*T*) curves are shown in Fig. 6:

(i) For 1, calculated dependencies point towards the full LS isomer to be the ground state up to *ca.* 20–210 K using the vibrational data obtained with both B3LYP* and B3LYP/D3. Around this temperature a crossing point of Δ*G* for all spin isomers is seen full HS isomer becomes that of the lowest Gibbs free energy at 315 K and above 400 K with B3LYP* and B3LYP/D3 vibrational data, respectively.

(ii) For 2, the fully LS isomer is predicted to be the ground state up to about 100 K with the vibrational data obtained with B3LYP*. Then the fully HS turns to be the one with lowest Gibbs free energy. The B3LYP/D3 predicts this to happen at *ca.* 65 K. For 2a, similarly full low spin switching temperatures of 65 and 85 K, respectively are predicted.

(iii) For 3, full HS isomer is predicted to be the ground state at 350 K if calculated with B3LYP/D3 derived vibrational data and at 350 K for the B3LYP* derived ones.

In all cases, this composite approach predicts the fully LS and HS chains to be the ground state depending on temperature. Thus our combined experimental and theoretical approach confirms that the heptanuclear 1D chain fragments of Fe(ii) complexes with 1,2,4-triazole-based ligands have a ferroelastic character and switch between the full LS and the full HS state on temperature change. This ferroelasticity is a result of a strong increase of the entropic effects for the HS chains with increasing temperature and the additional strain occurring for the spin isomer containing both spin centres.

**Fig. 7:**
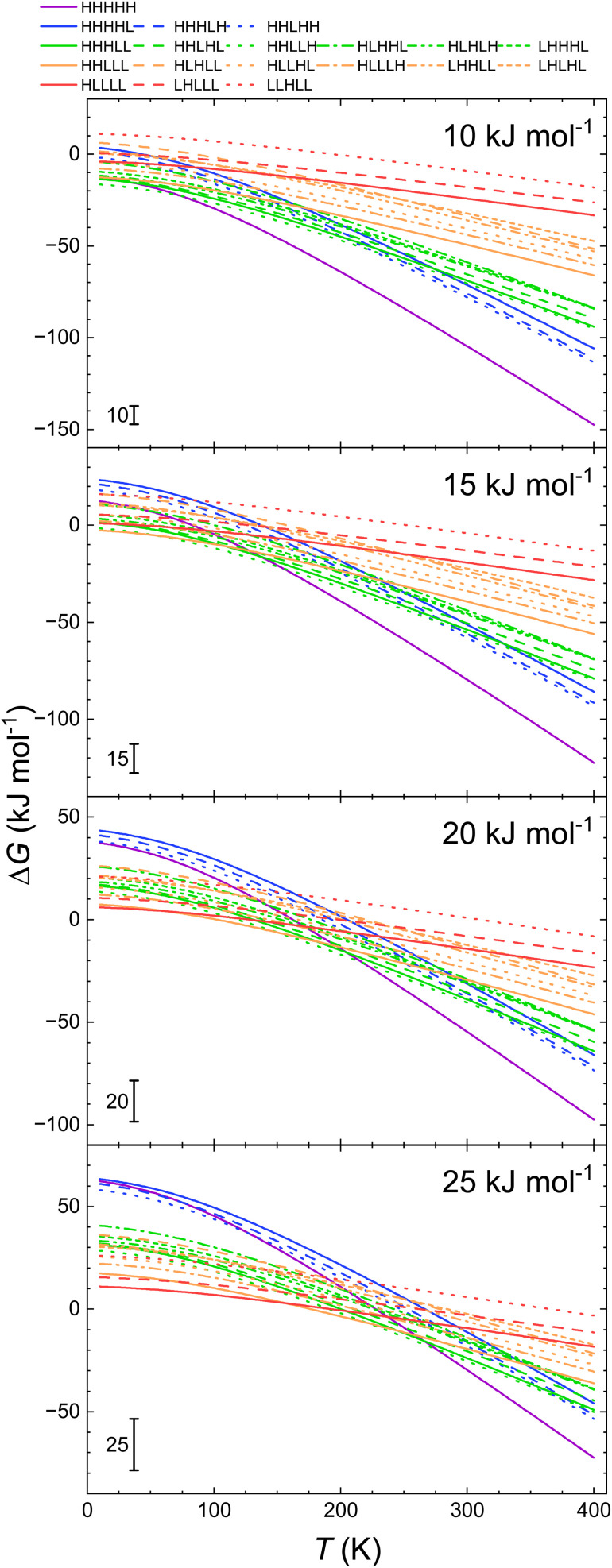
Temperature dependencies of the Gibbs free energy of HHHHHH spin isomer of 1 for different values of Δ*E*_ad._

**Fig. 8:**
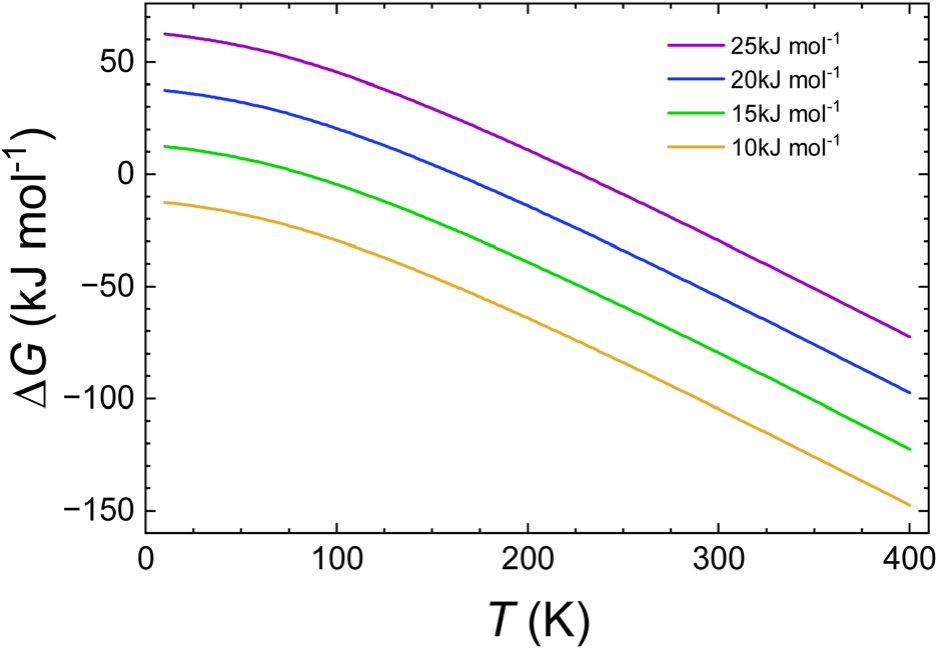
Temperature dependencies of the free enthalpy of HHHHH (relative LLLLL one) spin isomer of 1 for different values of Δ*E*_ad._

The above results allow the estimation of the effect of how the temperature dependencies of the free enthalpies of a spin isomer depend on the adiabatic electronic energies of the spin transition. Fig. 7 shows the dependencies obtained for 1 using eqn (3) with *E*_ad_ = Δ*H*_HL_ − Δ*H*_vib_(*T*_c_) as parameter. The diagrams show a very strong dependence of the predicted ground state on *E*_ad_. For example, the change from 20 kJ mol^−1^ to 10 kJ mol^−1^ shift the temperature at the which the fully HS state exhibits the lowest Gibbs free energy from *ca.* 320 K to *ca.* 270 K. Note that the difference of 10 kJ mol^−1^ may be compared to 12 kJ mol^−1^ difference of the conformer energies for ethane,^70^ thus corresponding to a very subtle changes of the molecular geometry. This effect is shown more clearly in Fig. 8 in which the relative free enthalpies of the full HHHHH model systems are shown as function of the temperature for different values of *E*. Last, but not least, the obtained temperature dependencies of Δ*G* obtained both on the basis of calculation and composite approach using the calorimetrically determined spin transition energies allow the estimation of the spin transition temperatures between fully LS and fully HS chains. The results are given in Table 8.

**Table 8:** Transition temperatures estimated on the basis of the Δ*G*(*T*) diagrams from Fig. 2–6

Model molecule	1	2	2a	3
*T*_c_ experimental (K)	390↑	343↑	332↑	347↑
	345↓	335↓	311↓	311↓

**Method**				
B3LYP*	Over 400 K	Over 400 K	Over 400 K	Over 400 K
B3LYP/geoB3LYP*	190 K	190 K	305 K	10 K
CAM-B3LYP/geoB3LYP*	160 K	150 K	320 K	120 K
B3LYP/D3	160 K	260 K	310 K	120 K
Cal/vib B3LYP*^a^	315 K	100 K	65 K	350 K
Cal/vib B3LYP/D3^a^	400 K	75 K	85 K	315 K

a Composite approach using the calorimetry data and vibrational data from DFT.

Interestingly, in spite of the obvious simplifications of the models (isolated molecules of finite size with no energetic and vibrational effects of crystal packing, within the harmonic approximation) give for systems 1 and 3 the reasonable estimation of the transition temperatures when the calorimetrically obtained values of spin transition energies are used.

### Thermal analyses

**Fig. 9:**
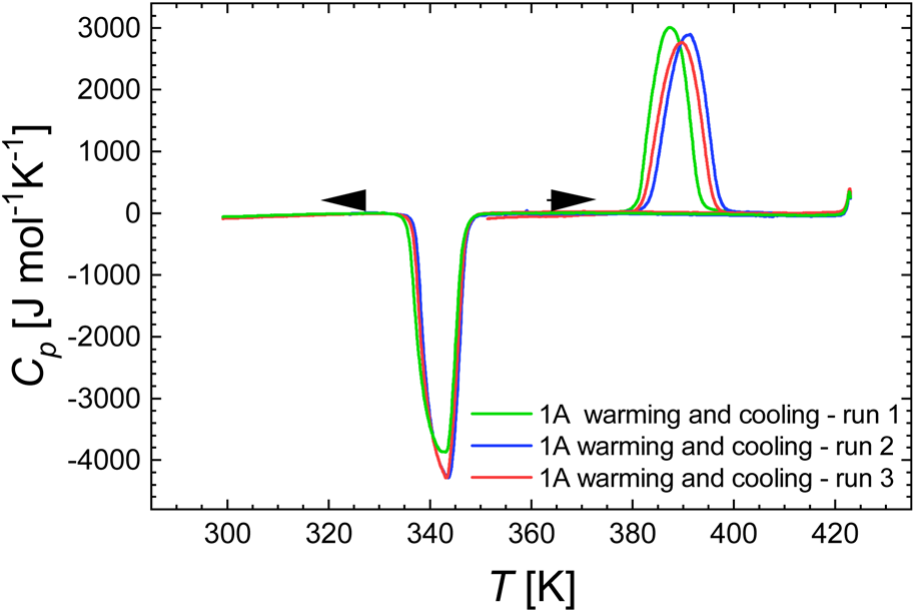
DSC measurements for [Fe(Htrz)_2_trz]BF_4_ (1) over the 300–423 K temperature range, at a scan rate of 10 K min^−1^, in cooling and warming modes, for three consecutive runs.

[Fe(Htrz)_2_trz]BF_4_ (1) was studied by DSC on both warming and cooling modes over the temperature range 300–425 K at a 10 K min^−1^. An endothermic peak is observed on warming at *T*_max_^↑^ = 387 K and an exothermic peak is recorded on cooling at *T*_max_^↓^ = 343 K (Fig. 9), in good agreement with the transition temperatures provided by Kröber *et al.*^59^

The energy and entropy associated with the spin transition were evaluated by considering the fraction of switching sites evaluated from Mössbauer spectroscopy (86%). This correction was not done in the original DSC report on this compound, which explains why corrected values are lower (compared to Δ*H*_HL_ = 27 kJ mol^−1^).^53^

**Fig. 10:**
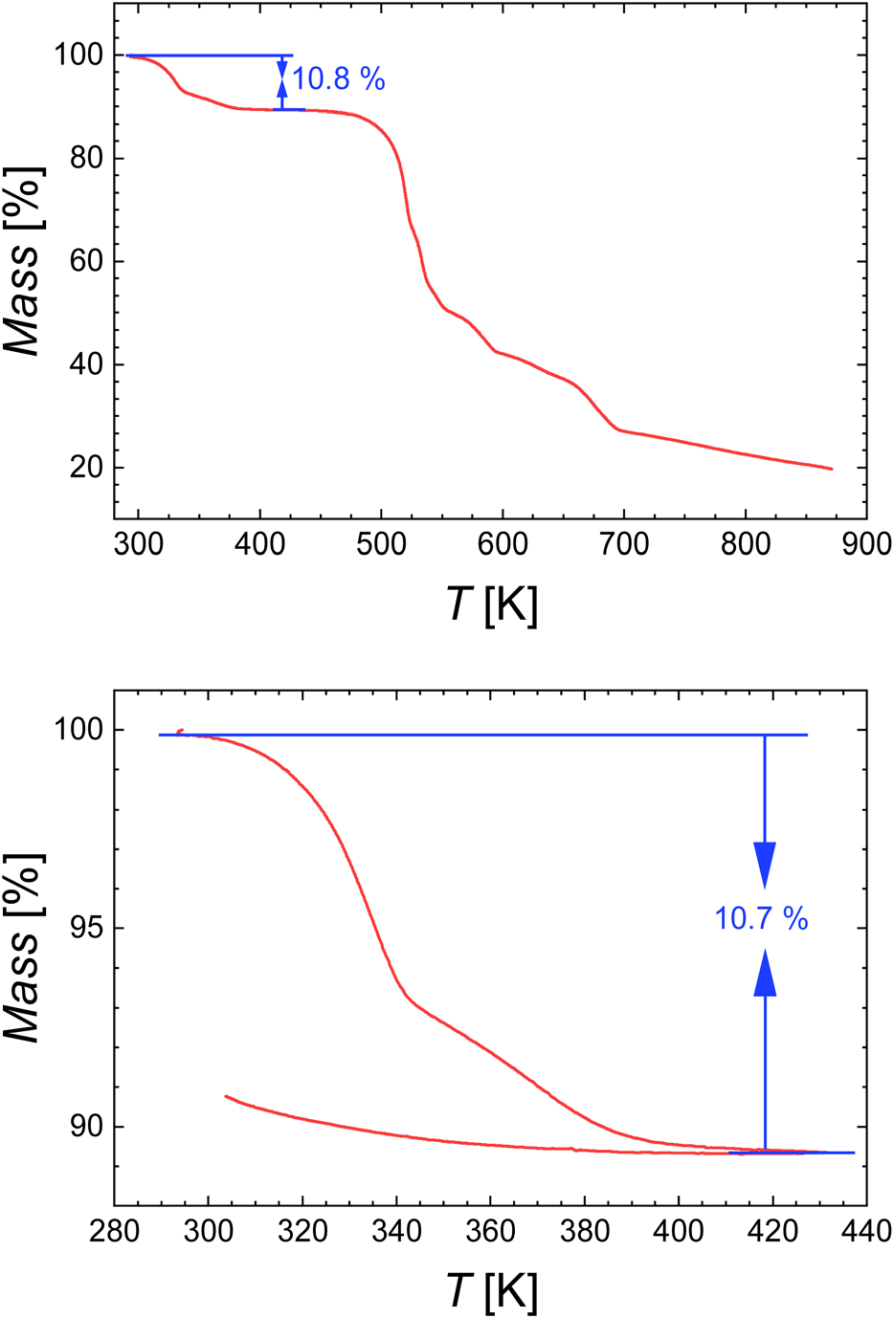
TGA curves of [Fe(NH_2_trz)_3_]Cl_2_·2.5H_2_O (2a) under N_2(g)_ (top) and under air (bottom).

TGA was undertaken under N_2(g)_ (100 mL min^−1^) from room temperature to 873 K with a heating rate of 5 K min^−1^ to determine water content (Fig. 10). The *ca.* 10.8% mass loss below 390 K was attributed to the loss of 2.5 water molecules, corresponding to the formula [Fe(NH_2_trz)_3_]Cl_2_·2.5H_2_O. Thermogravimetric measurements were also performed in air (100 mL min^−1^, 5 K min^−1^) over the temperature range of 298 K to 433 K. The mass loss before 420 K also corresponds to the loss of 2.5 water molecules (Fig. 10) no mass change was observed with decreasing temperature, indicating that the sample did not rehydrate in air, thus affording compound 2.

**Fig. 11:**
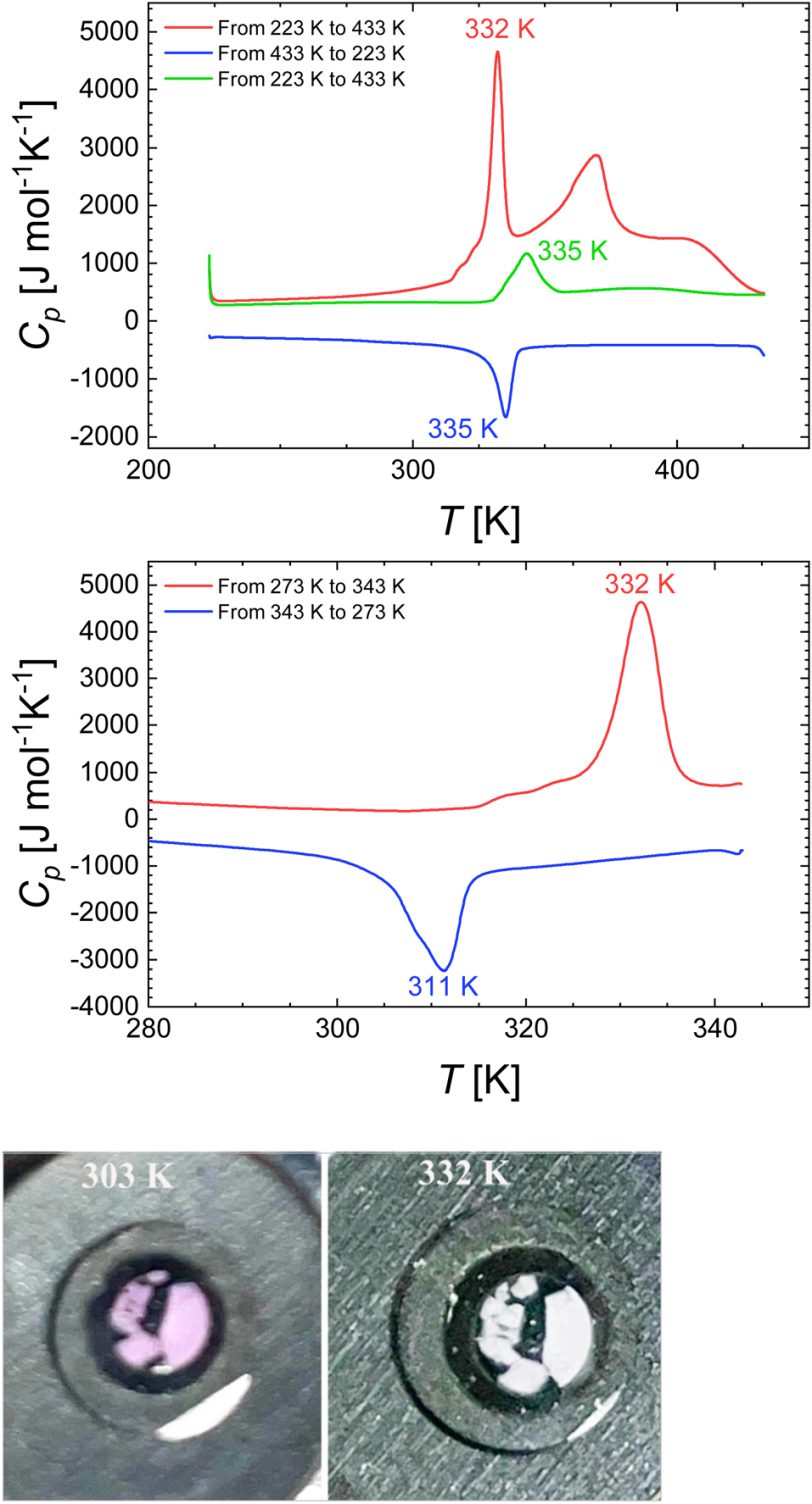
(Top) DSC curves of [Fe(NH_2_trz)_3_]Cl_2_·2.5H_2_O from 223 K to 433 K showing two peaks associated to SCO and to the removal of water molecules (red line) and [Fe(NH_2_trz)_3_]Cl_2_ during cooling (blue line) and heating modes (green line). (Middle) DSC curves of [Fe(NH_2_trz)_3_]Cl_2_·2.5H_2_O during cooling (blue line) and heating (red line) modes. (Bottom) Color change of [Fe(NH_2_trz)_3_]Cl_2_·2.5H_2_O from pink (303 K) to white (332 K).

DSC measurements were performed under a N_2(g)_ atmosphere with a heating rate of 5 K min^−1^ in the temperature range from 223 K to 433 K. Calorimetric data of 2a were first analysed within the temperature range of 223 K to 433 K, revealing two distinct endothermic peaks. A sharp peak at *T*_max_^↑^ = 332 K corresponding to the SCO of 2a from the LS state to the HS state (see colour change in Fig. 11), while a broader peak at 370 K is associated with the dehydration process (Fig. 11).

The warming mode was proceeded until complete dehydration observed at 433 K, according to thermogravimetry (Fig. 10). Subsequently, the dehydrated sample 2 was cooled to 223 K, during which an exothermic peak at *T*_max_^↓^ = 335 K was observed, indicating the transition from the HS state back to the LS state. During reheating, the transition from LS to HS was detected at 343 K, resulting in a hysteresis loop of 8 K width for 2. The energy and entropy changes were determined as Δ*H* = 8.93 kJ mol^−1^ and Δ*S* = 26.06 J K^−1^ mol^−1^, respectively. DSC characterization of 2a was carried out in the range of 273 K to 343 K, below the dehydration temperature (Fig. 10) to allow the missing thermodynamic parameters to be determined. Correspondingly, endothermic and exothermic peaks corresponding to the SCO were detected at 332 K and 311 K, respectively. This resulted in the formation of a hysteresis loop of 21 K width, which is much broader than that observed for 2. Such an increase in hysteresis width may be due to an increase of intermolecular interactions involving non coordinated water molecules with the NH_2_trz ligand and/or chlorine atoms in this system, as observed in the crystal structure of [Cu(NH_2_trz)_3_](NO_3_)_2_·H_2_O.^71^ The changes in energy and entropy were estimated as Δ*H* = 12.22 kJ mol^−1^ and Δ*S* = 39.38 J mol^−1^ K^−1^, respectively.

### Experimental and computational methods

#### Syntheses

[Fe(Htrz)trz]BF_4_ (1) was prepared at room temperature as a pink powder using an ethanol/water mixture of Fe(BF_4_)_2_·6H_2_O mixed with three equivalents of Htrz.^53^

[Fe(NH_2_trz)_3_]Cl_2_·2.5H_2_O (2a) was synthesized by adapting the reported synthesis.^54^ Dissolve 2 mmol (400 mg) of FeCl_2_·4H_2_O along with a small amount of ascorbic acid in water (10 mL). In a separate container, dissolve 20 mmol (1.68 g) of NH_2_trz in ethanol (15 mL). Stir the combined solutions at room temperature for 15 h. A white precipitate formed that gradually turned purple. The resulting precipitate was washed with ethanol and dried in air.

[Fe(NH_2_trz)_3_]Cl_2_ (2) was prepared *in situ*; warming 2a above at a temperature where full dehydration occurs (420 K), accurately determined by thermogravimetric analysis (TGA), see below.

#### Thermal analyses

DSC experiments were carried out on 1 in a He_(g)_ atmosphere using a PerkinElmer DSC Pyris 1 instrument equipped with a cryostat and operating down to 98 K. Aluminum capsules were loaded with 21.9 mg of sample and sealed. The heating and cooling rates were fixed at 10 K min^−1^. Temperatures and enthalpies were calibrated over the temperature range of interest (300–425 K) using the solid–liquid transitions of pure indium (99.99%). Enthalpy data were obtained by integration of the peaks using the PYRISTM DSC software 7.0 in specific heat *C*_p_ (J mol^−1^ K^−1^) format. The transition temperatures were derived by considering the maximum (*T*_max_) of the thermal anomalies.

Thermogravimetric analyses were undertaken on a Mettler Toledo TGA/SDTA 851e analyzer under N_2(g)_ (100 mL min^−1^) from room temperature to 873 K with a heating rate of 5 K min^−1^.

Differential scanning calorimetry (DSC) measurements on 2 and 2a were performed on a Mettler Toledo DSC 3 using a N_2(g)_ atmosphere with a heating rate of 5 K min^−1^ in the temperature range from 223 K to 433 K. Aluminium standard 40 mL capsules were loaded with 5.70 mg of sample and sealed. Temperatures and enthalpies were calibrated using pure indium and zinc. The specific heat capacity (*C*_p_) was determined using STARe software.

#### The models

Most of the modelling was performed with the heptanuclear models represented in Scheme 1. For 1 and 2 they were based on the heptameric models reported for some of the spin isomers in ref. 47*b* and 47*c*, respectively. The model 2a was built up after all spin isomers of 2 were optimised and then 12 molecules of water were introduced to form the CH⋯O(H_2_)···HC bridges between each two parallel arranged triazole ligands (see pdb files in SI). The chlorine anions were shifted on the rim of the chain in starting structures providing the stoichiometry of Fe_5_(H_2_O)_12_ for the inner switching iron centres in the heptanuclear units. This stoichiometry is between Fe(H_2_O)_2_ reported in literature^62^ and the Fe(H_2_O)_2.5_ derived from thermogravimetric data in this paper (*vide supra*). The model for 3 was based on the crystal structure of the LS isomer.^56^ While the calorimetric data for this complex are available for the anhydrous complex, the calculation were performed without water molecules. All system were modelled as the neutral molecules, with 2, 2a and 3 containing 14 anions, while the 1 one with 8 anions (there are six negatively charged triazole ligands in the structure of 1). Th structure of the HHHHH spin isomer of 3 was obtained on the basis of the optimised Zn_7_ analogue starting from the LLLLL structure with the further optimisation of the HHHHH model. As described previously^47^ all spin isomers containing both HS and LS centres in the inner pentanuclear unit were modelled on the basis of the optimised systems in which the HS Fe(ii) were replaced with Zn(ii). The Zn centres were subsequently replaced with Fe(ii), the stability of the wave function was then checked with the Gaussian stable = opt option and the molecule was further optimised for all-Fe centres. Such an approach yielded all spin isomers under consideration. The *H*_coop_ parameter was additionally estimated using the nonanuclear and undecanuclear models of four isomers, two with fully HS or LS inner Fe centres and two with one Fe centres of the other spin (*i.e.* the analogues of LLLLL, HHHHH, LLHLL and HHLHH, respectively). The model molecules were built up on the basis of the optimised heptanuclear models. In order to decrease the computational time the terminal HS centres with three coordinated waters were replaced with the Zn ones.

#### DFT methods

As stated previously, all optimisations and normal mode calculations were firstly performed with B3LYP* functional^63^ using Gaussian 16 package,^72^ Then the calculations were performed with B3LYP^66^ functional using the dispersion correction. For 1 and 2 the further optimization were performed with CAM-B3LYP^67^ functionals and the CEP-31G basis.^73^ Additionally, the PBEh functional was used for the above two systems.^74^ In order to check the dependence of the obtained *H*_coop_ parameters the relevant four isomers of 1, 2 and 2a were optimised with B3LYP, CAM-B3LYP and TPSS^75^ functionals with Grimme's D3 dispersion correction.^76^ The CEP-31G basis set was used for the former and the tzvp^77^ for the latter one. Also, the TPSSh functional^76,78^ was used together with the tzvp basis set. We used the default settings of Gaussian 16, optimising the internal coordinates with Berny algorithm^73^ (see https://gaussian.com/opt/ for details). The further details concerning the threshold values for optimisation, integral grid, the dispersion functions, *etc.* are given in SI.

## Conclusions

Four different 1D chain spin crossover Fe(ii) complexes with 1,2,4-triazole based ligands exhibiting sharp or hysteretic spin transitions have been modelled by DFT methods using heptanuclear model fragments. In each case 20 possible spin isomers were calculated varying the permutation of spin centres within the inner pentanuclear fragment. The B3LYP*/CEP-31G calculations revealed that each spin isomer has a distinct electronic and vibrational energy. Further energy calculations with the B3LYP and CAM-B3LYP functionals for the geometry of the spin isomers optimised with B3LYP* exhibit that each spin transition energy from the fully LS state to a given spin isomer may be considered as the sum of the functional-dependent quantity *E*_ad_ (derived from the calculated spin transition energy from the fully LS to the fully HS state) and the functional independent *H*_strain_. The latter is the result of the strain in the particular spin isomer due to short-range interactions between HS and LS centres. Calculations of the temperature dependence of the Gibbs free energy change on the spin transition from the LLLLL isomer to all 19 spin isomers were performed. These results point towards ferroelastic properties of the isolated 1D chains under study suggesting a direct switch between the fully LS and HS isomers at a sufficiently low value of *E*_ad_. It was also found that short-range interactions within the chain affect even centres that are 1.6 nm apart (*i.e.* with 3 Fe(ii) centres in between) in an HS matrix with LS defects. The corresponding interactions in the LS matrix occur at significantly lower distances (1.1 nm for 3). The comparison between obtained results and the experimental finding may be summarized in three points: (i) all four systems under study reveal the sharp transitions indicating the ferroelastic character of both short- and long-range interactions. Our results show that the ferroelastic properties are present in the relatively short fragments of chains of these molecules. Additionally, the most destabilised spin isomers seem to be those with antiferroelastic structure. (ii) Also for complexes 2a and 3 the effect of elongation of the bonds of the LS centres by the HS neighbours and the shortening of the HS centres by the LS neighbours is predicted with DFT methods. This effect was previously predicted and experimentally observed with nuclear inelastic scattering for 1 and 2.

(iii) The composite approach combining the DFT calculated *H*_strain_ parameters and vibrational properties with the calorimetrically determined spin transition energies gives the reasonable estimate of the experimentally determined spin transition energies for two of the four studied compounds.

We believe that the above results may be helpful in the further development of the phenomenological models.

## Author contributions

JAW, HP and VS conceived the paper, MD, XL and YG performed and evaluated the calorimetric measurements, JAW performed the DFT calculations, KG, TH and JAW calculated the thermodynamic functions, XL synthesized the complexes, JAW, YG, HP and VS wrote the paper.

## Conflicts of interest

There are no conflicts to declare.

## Supplementary Material

RA-015-D5RA03472H-s001

RA-015-D5RA03472H-s002

## Data Availability

The calculated relative energies are given in text. In SI the calculated optimised structures are given as xyz file and harmonic frequencies for each modelled systems are included. Also the computational results and details, and details of thermochemical measurements are included. See DOI: https://doi.org/10.1039/d5ra03472h.
