# Supplementary material for: Combination of density functional theory and calorimetry reveals the microscopic nature of spin state switching in 1D Fe(ii) spin crossover complexes
Source: RSC Adv. 2025 Sep 9;15(38):32009–30. doi: 10.1039/d5ra03472h (PMC12419163; doi:10.1039/d5ra03472h)
Supplement: RA-015-D5RA03472H-s002 [file RA-015-D5RA03472H-s002.pdf]

Supplementary Information (SI) for RSC Advances.
This journal is © The Royal Society of Chemistry 2025

ESI.

Combination of density functional theory and calorimetry reveals the microscopic nature of spin state switching in 1D Fe(II) spin crossover complexes

Juliusz A. Wolny[a], Xiaochun Li[b], Marinela Dîrtu[b], Konstantin Gröpl[a], Tim Hochdörffer[a], Hauke Paulsen[c], Yann Garcia[b] and Volker Schünemann[a]

Table of contents.

S1. Details of the Gaussian calculations.

S2. Graphic representations of the calculated electronic energy of different spin isomers calculated with B3LYP*/CEP-31G.

Figure S1. Calculated (B3LYP*/CEP-31g) relative electronic energies of the spin isomers of **1**, E_{el} of HLLLLLH one assumed to be zero.

Figure S2. Calculated (B3LYP*/CEP-31g) relative electronic energies of the spin isomers of **2**, E_{el} of HLLLLLH one assumed to be zero.

Figure S3. Calculated (B3LYP*/CEP-31g) relative electronic energies of the spin isomers of **2a**, E_{el} of HLLLLLH one assumed to be zero.

Figure S4. Calculated (B3LYP*/CEP-31g) relative electronic energies of the spin isomers of **3**, E_{el} of HLLLLLH one assumed to be zero.

S3. Anion-ligand and anion-solvent (2a) interactions.

Figure S5. Selected intermolecular distances in the B3LYP* optimised structures of HS (top) and LS (bottom) spin isomers of **1**. Only the contacts shorter than 3 Å were shown. Note that all contacts are shorter for the LS isomer.

Figure S6. Selected intermolecular distances in the B3LYP* optimised structures of HS (top) and LS (bottom) spin isomers of **2**. Only the contacts shorter than 3 Å were shown. Note that all contacts are shorter for the LS isomer.

Figure S7. Selected intermolecular distances in the B3LYP* optimised structures of HS (left) and LS (right) spin isomers of **2a**. Only the contacts shorter than 3 Å were shown. Note that contrary to dehydrated system **2** no short contacts between Cl^- anion and triazole H(C) occur. Instead, the water oxygen binds between two parallel triazole rings forming the H_2O-H(C) hydrogen bonds. In this case they are shorter for the HS state. The chlorine atoms are involved in contacts with water and amino hydrogens.

Figure S8. Selected intermolecular distances in the B3LYP* optimised structures of HS (left) and LS (right) spin isomers of **3**. Only the contacts shorter than 3 Å were shown. Note the shortening of the O(NO_3^-)-H(C) distances and lengthening of the O(NO_3^-)-H(NH_2) ones on going from HS to LS state.

S4. Short interaction parameters other than H_{coop}.

Figure S9. Graphic representation of the parameters reflecting the effect of the nearest neighbour "defect" on the spin transition energies.

Figure S10. Graphic representation of the parameters reflecting the effect two nearest neighbour "defect" on the spin transition energies.

Figure S11. Graphic representation of the parameters reflecting the effect of next-to-nearest neighbour "defect" on the spin transition energies.

Figure S12. Graphic representation of the parameters reflecting the effect of two next-to-nearest neighbours "defect" on the spin transition energies.

Figure S13. Graphic representation of the parameters reflecting the effect of the neighbour separated by two Fe-centres on the spin transition energies.

Figure S14. Graphic representation of the parameters reflecting the effect of the neighbour separated by three Fe-centres on the spin transition energies.

Table S1. The values of different interaction parameters for the model systems **1**-**3**.

S5. Dependence of Hcoop and Hstrain on the applied DFT tools.

Table S2. Calculated H_{coop} values for the **1**, **2** and **2a** systems optimised with the different DFT methods.

Table S3. Calculated electronic energies and H_{strain} parameters obtained with B3LYP/D3 CAM-B3LYP/D3 and PBEh functionals for **2**.

Table S4. Calculated electronic energies and H_{strain} parameters obtained with B3LYP/D3 for **2a.** Zero-point corrections are given. The ZPE and H_{strain} values are also given for B3LYP*.

Table S5 Calculated electronic energies (kJ·mol^{-1}, B3LYP/D3), zero-point energy differences and H_{strain} (kJ·mol^{-1}) for **3** relative to those for LLLLL isomer.

Table S6. Zero-point corrections for **1** obtained with different functionals**.**

Table S7. Comparison of the mean Fe-N values for the LLLLL and HHHHH and the spin isomers revealing the largest strain obtained for the complexes under study for **2a** (very close values of H_{strain} for calculations with B3LYP* and B3LYP/D3) and **1** and **3** (larger discrepancy of respective H_{strain} values).

S6. ***Vibrational entropy as function of the applied DFT functional.***

Figure S15. Comparison of the calculated temperature dependence of vibrational entropy calculations of the spin transition from the LLLLL spin isomers for the model systems optimised with B3LYP/D3 (left) and B3LYP* (right).

Table S8. Vibrational entropy (J· K^{-1} ·mol^{-1}) at 300 K ranges for different spin isomers containing the same amount of HS centres for the modelled systems.

Figure S16. Comparison of the calculated temperature dependence of vibrational entropy calculations of the spin transition from the LLLLL spin isomers for **1** optimised with B3LYP/D3 (top left), B3LYP (top right), CAM-B3LYP/D3 (middle, left), CAM-B3LYP (middle, right), PBEh (bottom, left).

Figure S17. Comparison of the calculated temperature dependence of vibrational entropy calculations of the spin transition from the LLLLL spin isomers for the model system **2** optimised with B3LYP/D3 (left), and CAM-B3LYP/D3 (right).

S7. ΔG(T) dependencies for **1** and **2** calculated after geomtry optimisations with B3LYP and CAM-B3LYP with and without dispersion correction.

Figure S18. Temperature dependence of ΔG of the LLLLL to HHHHH transition for **1**. Top: calculated with B3LYP/D3 (left) and without dispersion correction (right). Bottom: calculated with CAM-B3LYP/D3 (left) and without dispersion correction

Figure S19. Temperature dependence of ΔG of the LLLLL to HHHHH transition for **2**. Left: calculated with B3LYP/D3 (left). Rights: calculated with CAM-B3LYP/D3 (left).

S8. Remarks on the completeness of spin transition for the calorimetrically measured samples of $[Fe(NH_2trz)_3]Cl_2$ and ($[Fe(NH_2trz)_3]Cl_2 \cdot 2H_2O$).

S1. Details of the Gaussian calculations with the default setting.

Thresholds for the SCF

Threshold for energy: 10^{-6} Hartree

Threshold for density: 10^{-8}

Threshold for gradient: 10^{-2}

Thresholds for the geometry optimisation.

Maximum Force: 0.00045 Hartrees/Bohr (or Radian)

RMS Force: 0.0003 Hartrees/Bohr (or Radian)

Maximum Displacement: 0.0018 Bohr (or Radian)

RMS Displacement: 0.0012 Bohr (or Radian)

Relaxation performed with internal coordinates.

Cartesian basis sets were used.

Tolerances for the Schwarz screening for the Coulomb integrals and electron density Coulomb Integral Screening, 10^{-8}

Electron Density Screening tolerance.

While not directly set as a tolerance, it's indirectly controlled by the SCF convergence threshold.

Damping function of dispersion correction

$$f_{d.n}(r_{AB}) = \frac{1}{1 + 6\left(\frac{r_{AB}}{s_{r,n}R_0^{AB}}\right)^{-\alpha_n}},$$

See S. Grimme, J. Antony, S. Ehrlich, H. Krieg H *J. Chem. Phys.* 2010, **132**, 154104 for details

Parameters	S6	SR6	S8
B3LYP	1.0000	1.2610	1.7030
CAM–B3LYP	1.0000	1.3780	1.2170
TPSSTPSS	1.0000	1.1660	1.1050

Level shifting for the unoccupied elements of the Fock matrix is not used in the final converged SCF value.
The SVD algorithm is used to check for possible linear dependencies in the basis set and the threshold defaults to 10^{-6}.

Integration:
Integration grid a pruned (99,590) grid. 99-point radial quadrature and 590-point Lebedev angular quadrature. Stratmann-Scuseria weight scheme which documented in R.E. Stratmann, G. E. Scuseria, Michael J. Frisch *Chem. Phys. Lett.* 257, 213 (1996)

S2. Graphic representation of the calculated electronic energy of different spin isomers calculated with B3LYP*/CEP-31G.

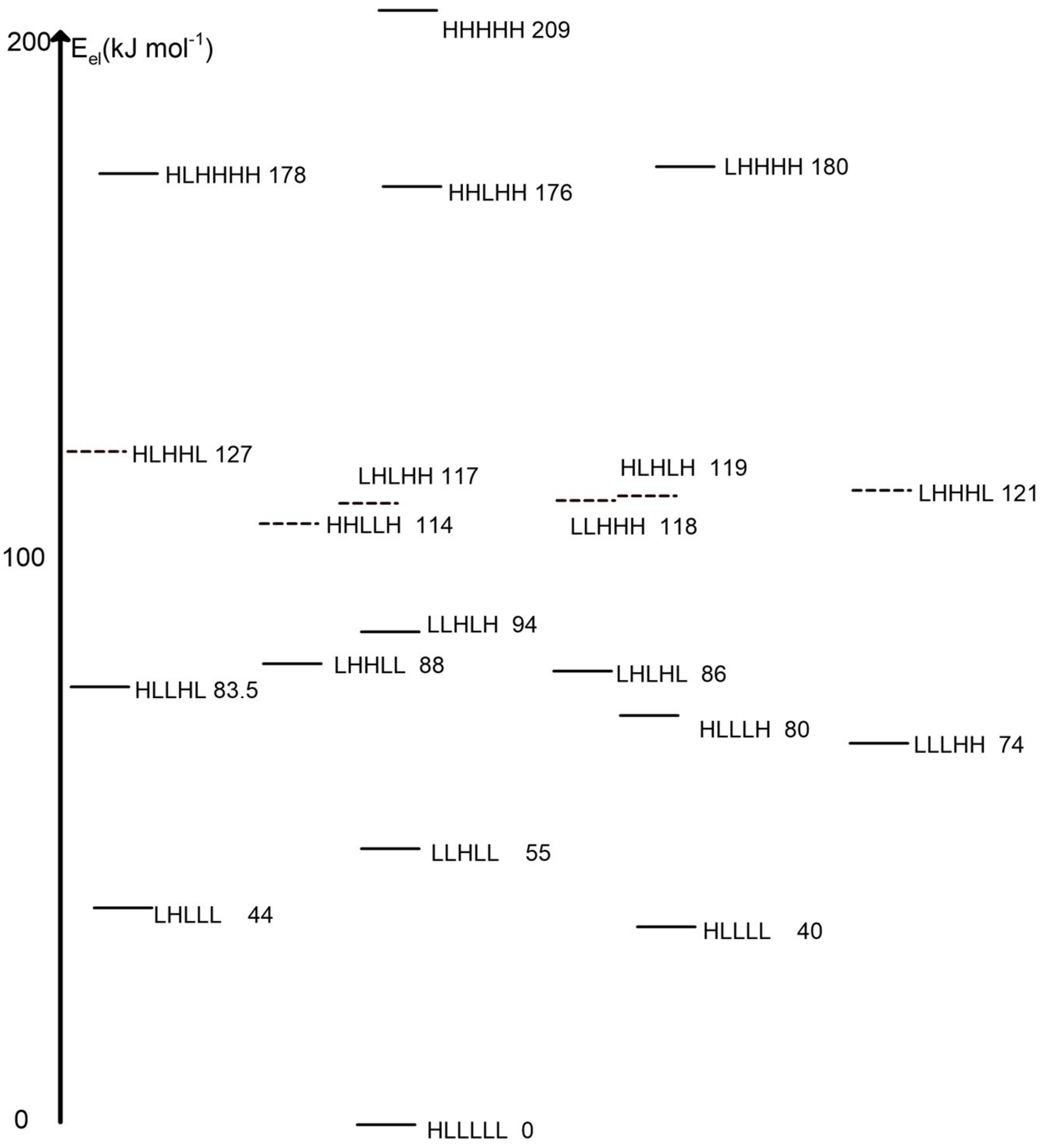

Figure S1. Calculated (B3LYP*/CEP-31G) relative electronic energies (kJ mol^{-1}) of the spin isomers of **1**, E_{el} of HLLLLLH one assumed to be zero.

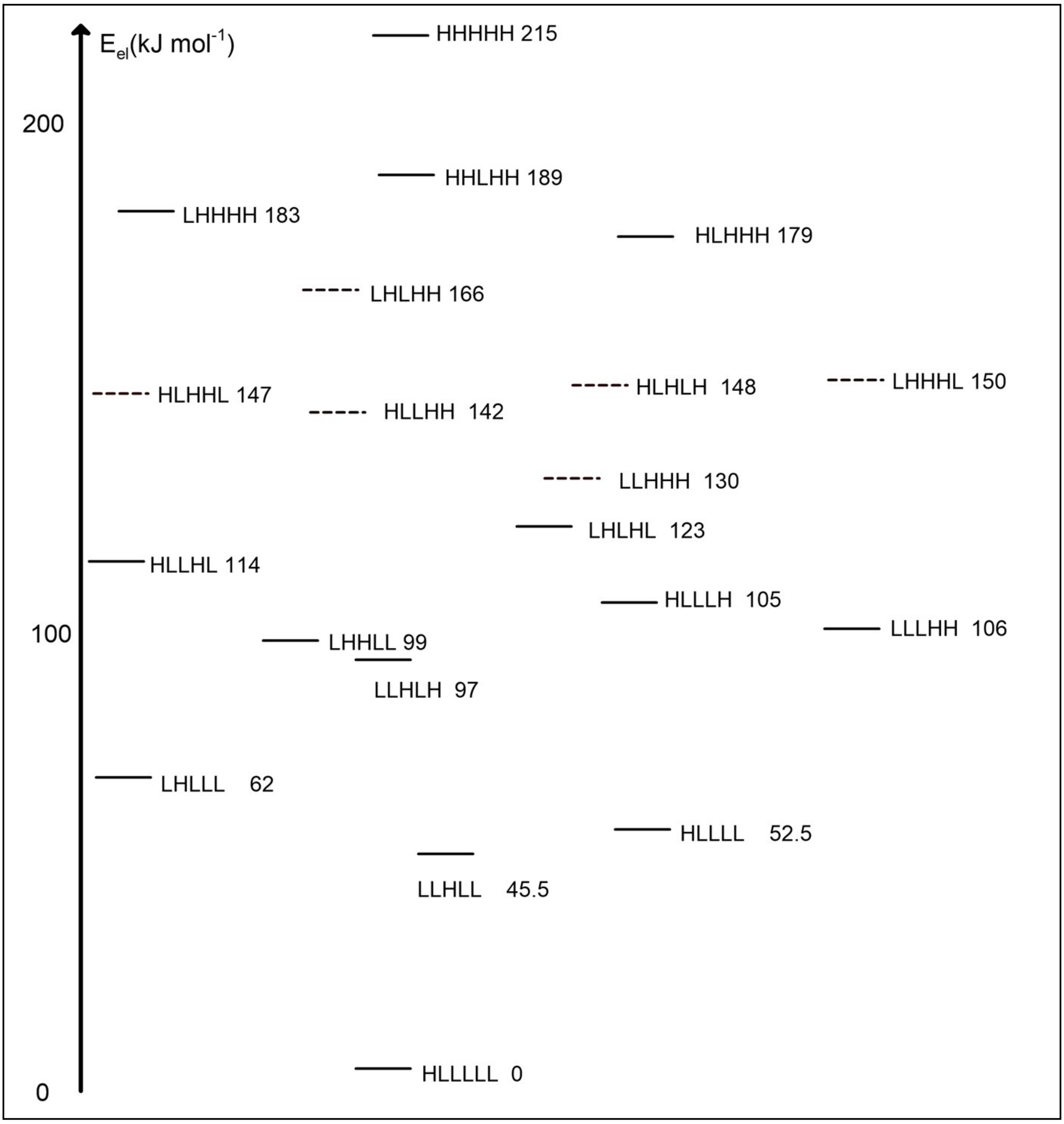

Figure S2. Calculated (B3LYP*/CEP-31G) relative electronic energies (kJ mol^{-1}) of the spin isomers of **2**, E_{el} of HLLLLLH one assumed to be zero.

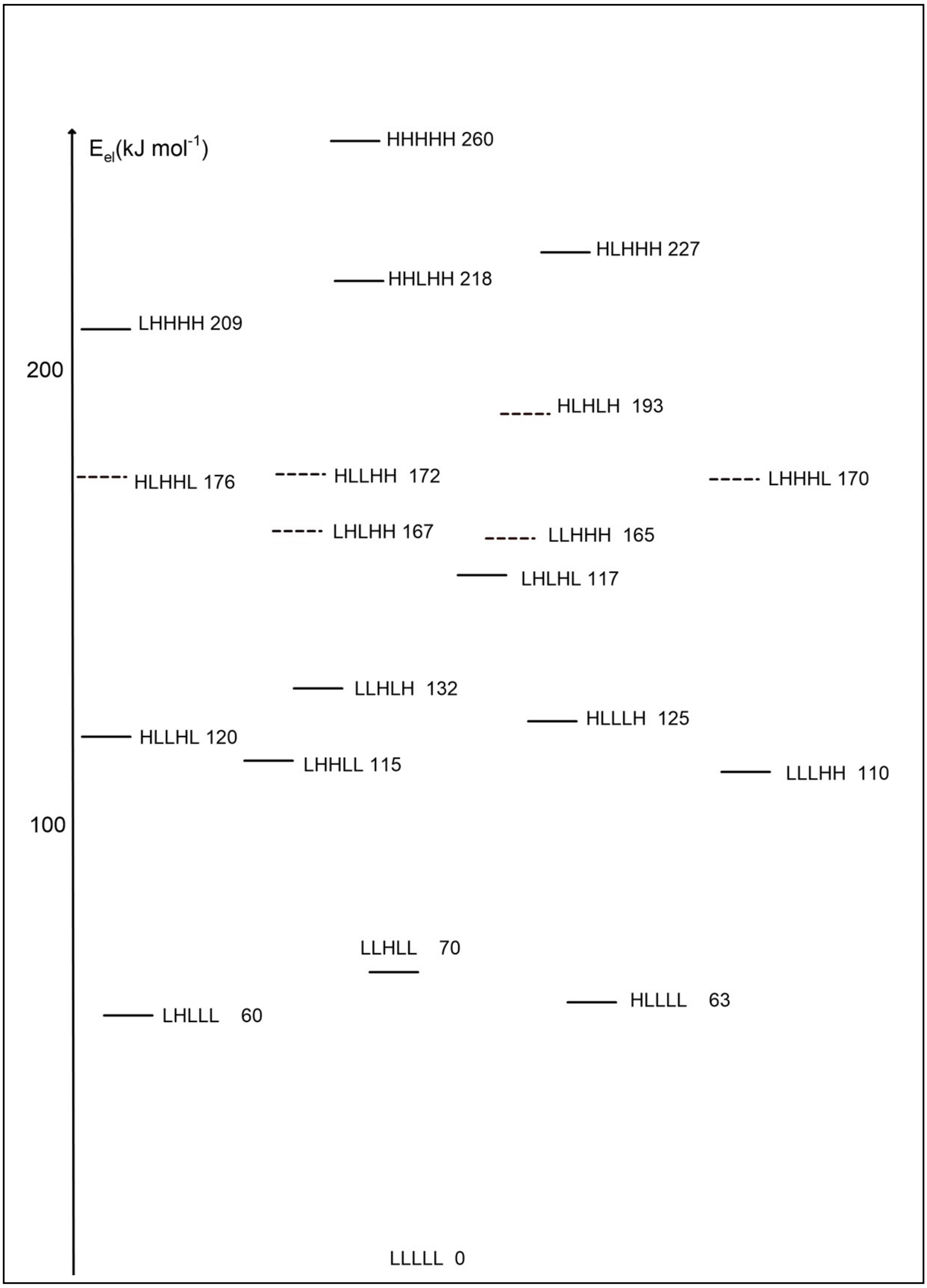

Figure S3. Calculated (B3LYP*/CEP-31G) relative electronic energies of the spin isomers of **2a**, E_{el} of LLLLL one assumed to be zero.

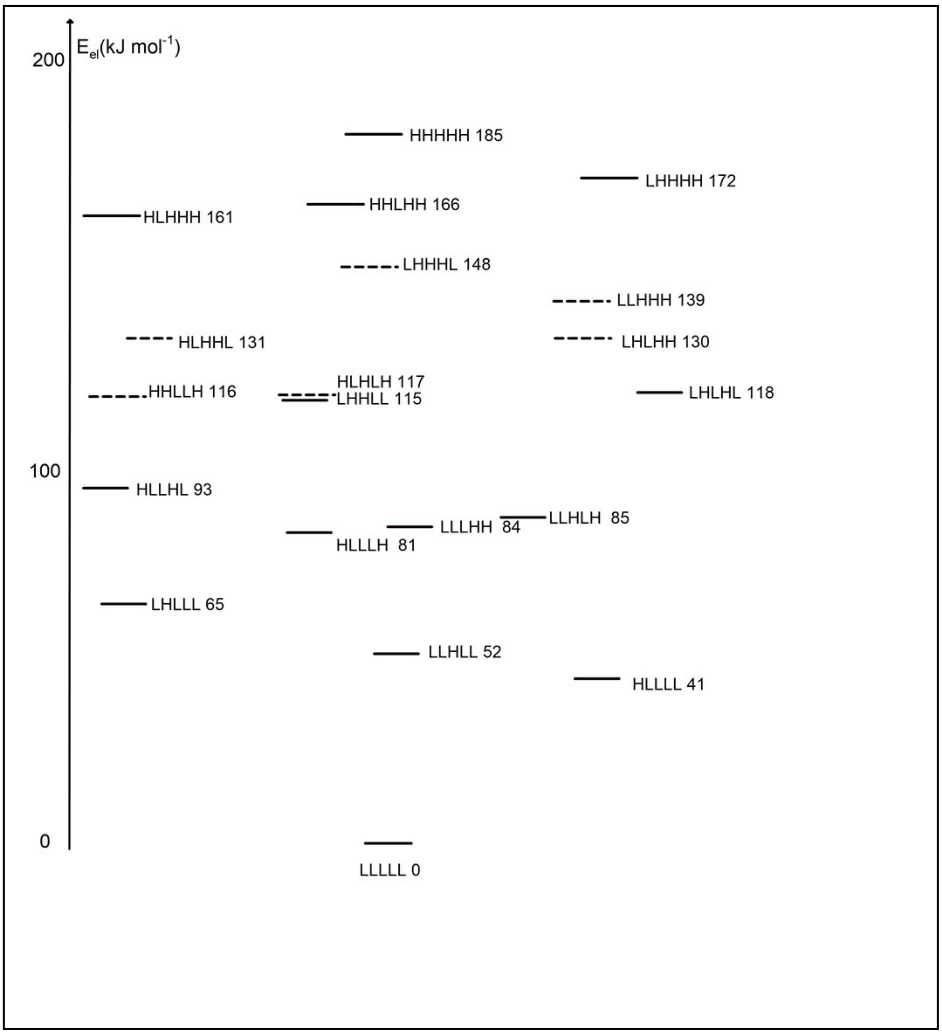

Figure S4. Calculated (B3LYP*/CEP-31G) relative electronic energies of the spin isomers of **3**, E_{el} of LLLLL one assumed to be zero.

S3. Anion-ligand and anion-solvent (2a) interactions.

Selected interatomic distances of the intermolecular contacts are shown for the model molecules **1**-**3**.

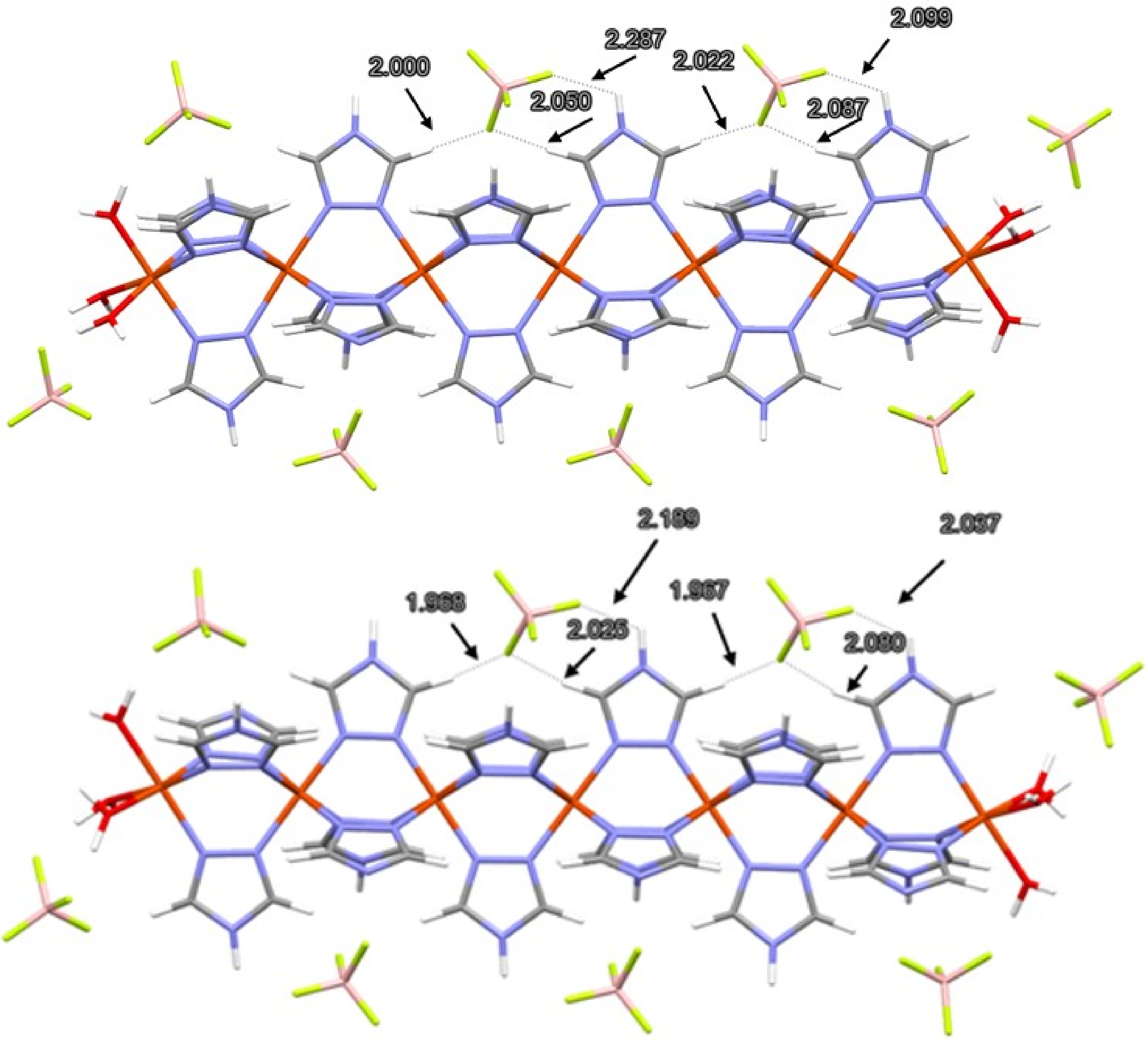

Figure S5. Selected intermolecular distances in the B3LYP* optimised structures of HS (top) and LS (bottom) spin isomers of **1**. Only the contacts shorter than 3 Å were shown. Note that all contacts are shorter for the LS isomer.

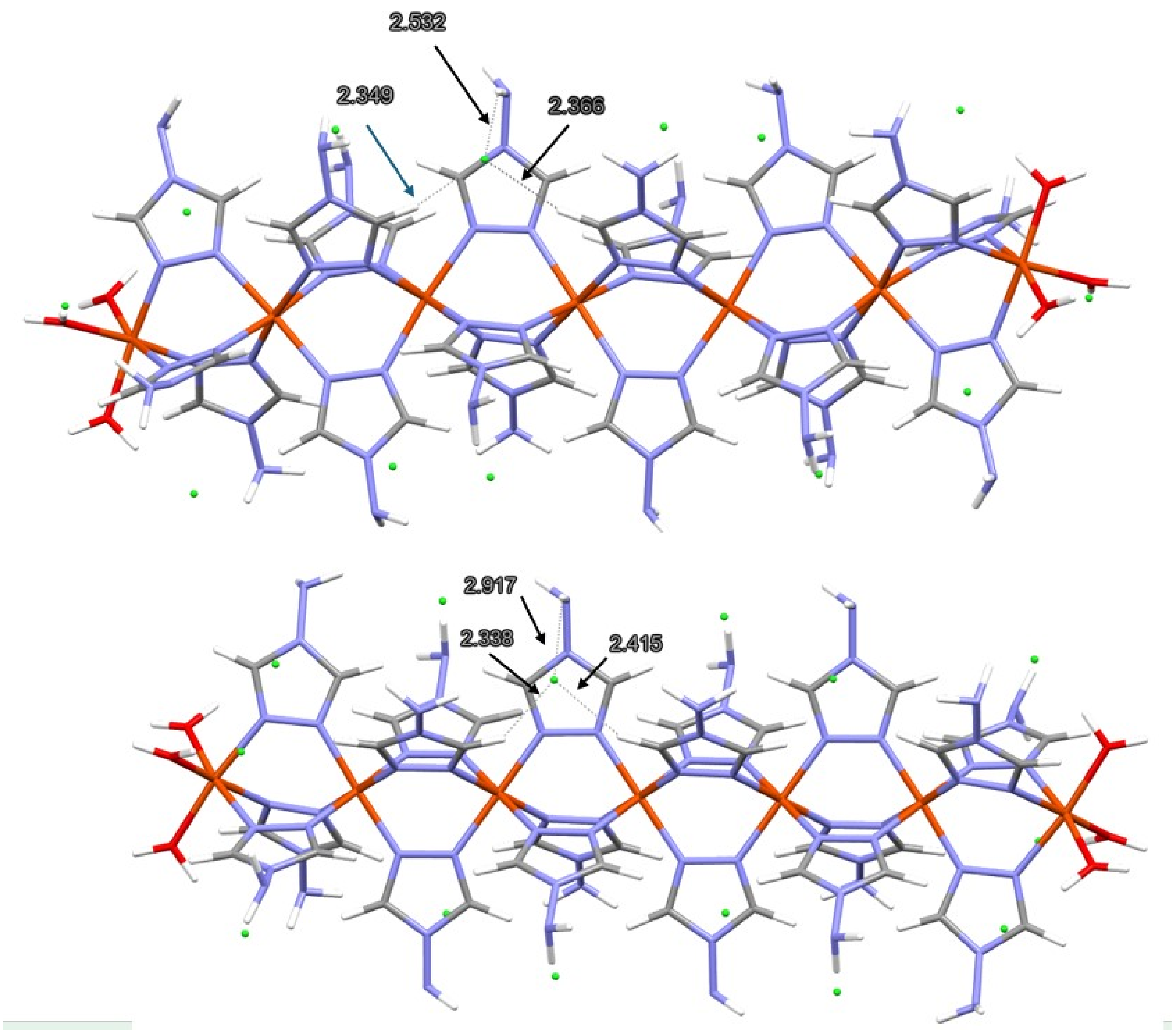

Figure S6. Selected intermolecular distances in the B3LYP* optimised structures of HS (top) and LS (bottom) spin isomers of **2**. Only the contacts shorter than 3 Å were shown. Note that all contacts are shorter for the LS isomer.

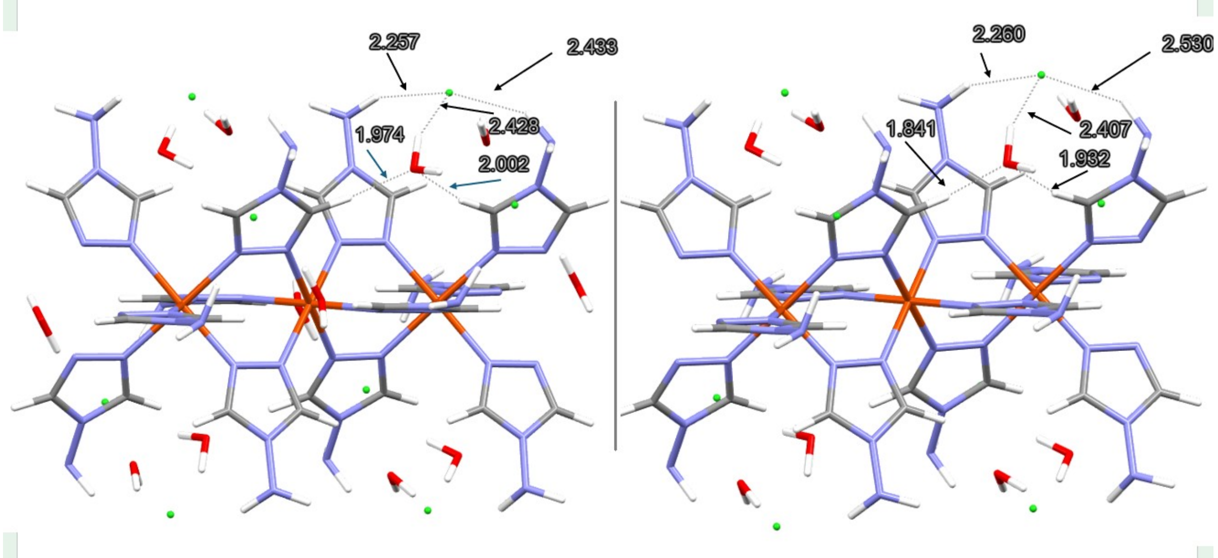

Figure S7. Selected intermolecular distances in the B3LYP* optimised structures of HS (left) and LS (right) spin isomers of **2a**. Only the contacts shorter than 3 Å were shown. For sake of clarity only the central trinuclear fragment of the chain is shown. Note that contrary to dehydrated system **2** no short contacts between Cl^- anion and triazole H(C) occur. Instead the water oxygen binds between two parallel triazole rings forming the H_2O-H(C) hydrogen bonds. The chlorine atoms are involved in contacts with water and amino hydrogens.

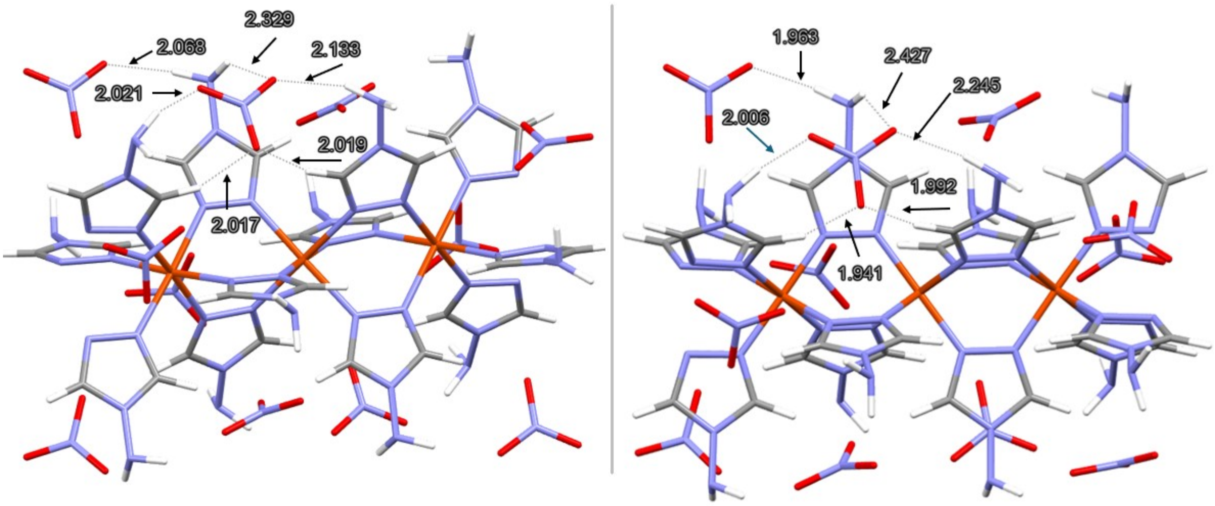

Figure S8. Selected intermolecular distances in the B3LYP* optimised structures of HS (left) and LS (right) spin isomers of **3**. Only the contacts shorter than 3 Å were shown. For sake of clarity only the central trinuclear fragment of the chain is shown. Note the shortening of the O(NO_3^-)-H(C) distances on going from HS to LS state.

S4. Short interaction parameters other than H_{coop}

Apart from H_{coop} several other parameters may be defined, that reflect the dependence of the LS -> HS spin transition energies for a given centre on the spin state of a given neighbour/neighbours. Table S1 lists the results such parameters for the systems under study. In this Table, the terms LS or HS matrix relates to a spin-state that prevails in the inner pentanuclear core of the models.

The parameters:

- $^{LS}H_{1D}$ and $^{HS}H_{1D}$ – describe the influence of spin of the nearest neighbour on the LS->HS spin transition of the Fe(1) centre in the predominantly LS or HS matrix, respectively. A visualization is shown in Fig S9.

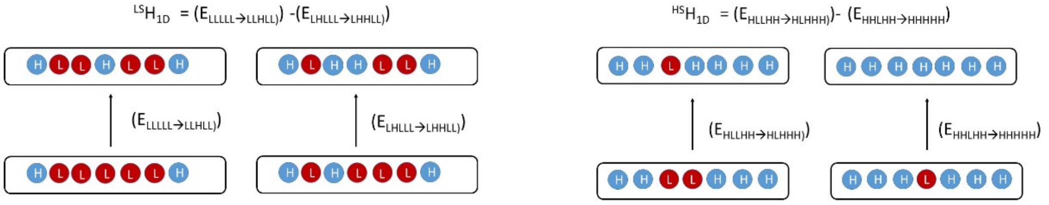

Figure S9. Graphic representation of the parameters reflecting the effect of the nearest neighbour "defect" on the spin transition energies.

- $^{LS}H_{2D}$ and $^{HS}H_{2D}$ – describe the influence of spin of the two nearest neighbours on the LS->HS spin transition of the Fe(1) centre in the predominantly LS or HS matrix, respectively. A visualization is shown in Fig. S10.

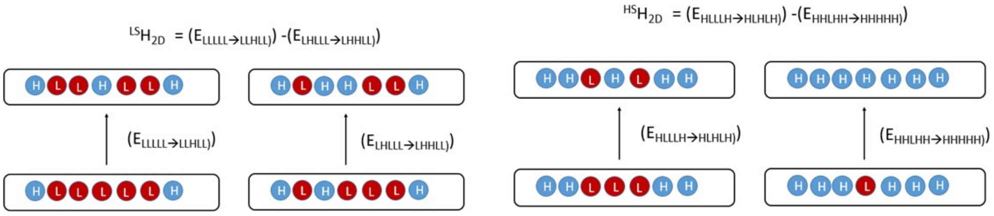

Figure S10. Graphic representation of the parameters reflecting the effect of a two nearest neighbour "defect" on the spin transition energies.

- $^{LS}H_{ND}$ and $^{HS}H_{ND}$ – describe the influence of spin of one next-to-nearest neighbour on the LS->HS spin transition of the Fe(1) centre in the predominantly LS or HS matrix, respectively. A visualization is shown in Fig. S11.

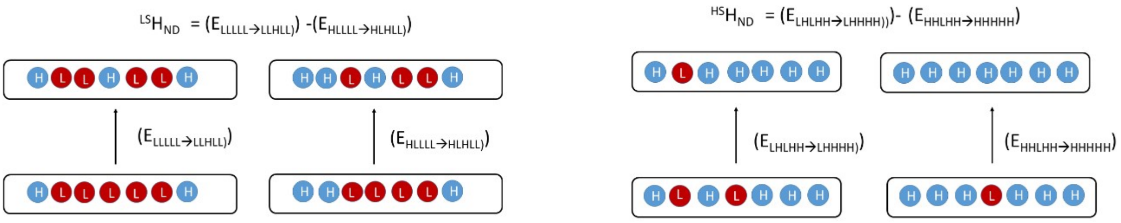

Figure S11. Graphic representation of the parameters reflecting the effect of next-to-nearest neighbour "defect" on the spin transition energies.

- $^{LS}H_{N2D}$ and $^{HS}H_{N2D}$ – describe the influence of spin of two next-to-nearest neighbour on the LS->HS spin transition of the Fe(2) centre in the predominantly LS or HS matrix, respectively. A visualization is shown in Fig. S12.

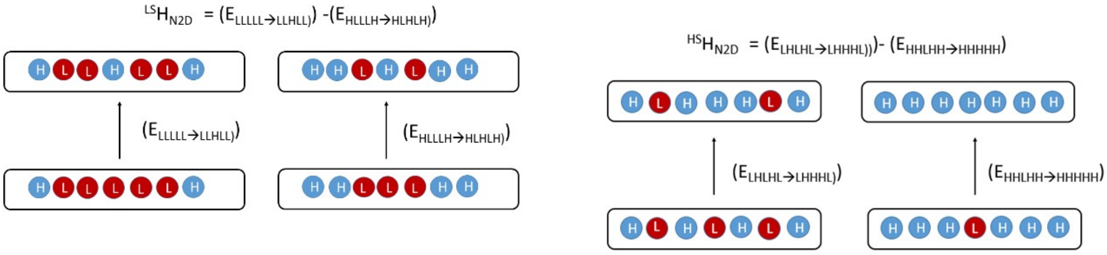

Figure S12. Graphic representation of the parameters reflecting the effect of two next-to-nearest neighbours "defect" on the spin transition energies.

- $^{LS}H_{3D}$ and $^{HS}H_{3D}$ – describe the influence of spin of the neighbour separated by two Fe-centres on the LS->HS spin transition of the Fe(2) centre in the predominantly LS or HS matrix, respectively. A visualization is shown in Fig. S13.

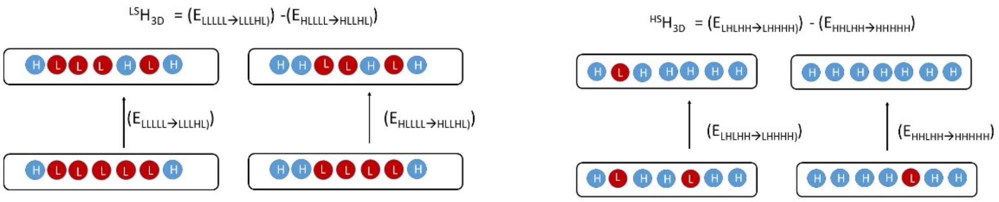

Figure S13. Graphic representation of the parameters reflecting the effect of the neighbour separated by two Fe-centres on the spin transition energies.

- $^{LS}H_{4D}$ and $^{HS}H_{4D}$ – describe the influence of spin of the neighbour separated by three Fe-centres on the LS->HS spin transition of the Fe(3) centre in the predominantly LS or HS matrix, respectively. A visualization is shown in Fig. S14.

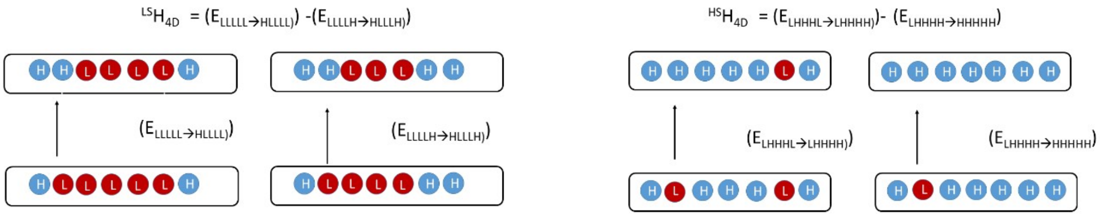

Figure S14. Graphic representation of the parameters reflecting the effect of the neighbour separated by three Fe-centres on the spin transition energies.

Matrix	Effect			1	2	2a	3
LS	Nearest neighbour defect	$^{LS}H_{1D}$	$(E_{LLLLL\rightarrow LLHLL)})$ - $(E_{LHLLL\rightarrow LHHLL)})$	11	8.5	14.5	1.5
HS		$^{HS}H_{1D}$	$(E_{HLLHH\rightarrow HLHHH)})$- $(E_{HHLHH\rightarrow HHHHH})$	31	8.0	12	25.5
LS	Two nearest neighbours defects	$^{LS}H_{2D}$	$(E_{LLLLL\rightarrow LLHLL)})$- $(E_{LHLHL\rightarrow LHHHL})$	20	17.5	17	22
HS		$^{HS}H_{2D}$	$-(E_{HLLLH\rightarrow HLHLH)})$- $(E_{HHLHH\rightarrow HHHHH})$	2	2.0	12	22
LS	One next-to-nearest neighbours defect	$^{LS}H_{ND}$	$(E_{LLLLL\rightarrow LLHLL)})$- $(E_{HLLLL\rightarrow HLHLL})$	1	1.0	1	8
HS		$^{HS}H_{ND}$	$(E_{LHLHH\rightarrow LHHHH)})$- $(E_{HHLHH\rightarrow HHHHH})$	30	-9.0	0	24
LS	Two next-to-nearest neighbours defects	$^{LS}H_{N2D}$	$(E_{LLLLL\rightarrow LLHLL})$ - $(E_{HLLLH\rightarrow HLHLH)})$	16	2.5	2	15
HS		$^{HS}H_{N2D}$	$(E_{LHLHL\rightarrow LHHHL)})$ $(E_{HHLHH\rightarrow HHHHH})$	2	2.0	12	11
LS	Defect separated by two Fe-centres from the switching centre	$^{LS}H_{3D}$	$(E_{LLLLL\rightarrow LLLHL})$- $(E_{HLLLL\rightarrow HLLHL)})$	0.5	0.5	0.5	13
HS		$^{HS}H_{3D}$	$(E_{HLHHL\rightarrow HHHHL})$ $-(E_{LHHHH\rightarrow HHHHH)})$	22	-3.0	0	16
LS	Defect separated by three Fe-centres from the switching centre	$^{LS}H_{4D}$	$(E_{LLLLL\rightarrow HLLLL)})$ - $(E_{HLLLL\rightarrow HLLLH})$	0	0.0	0.1	2.5
HS		$^{HS}H_{4D}$	$E(_{LHHHL->HHHHL)})$- $(E_{LHHHH->HHHHH})$	30	0.0	-12	11

Table S1. The values of different interaction parameters for the model systems **1**-**3**.

H_{1D} – For the complex **1**, the LS matrix the spin switching energy of Fe(1) is 55 kJ mol^{-1}. For the LS matrix with one HS defect at Fe(2) atom the spin switching energy of Fe(1) (i.e. LHLLL to LHHLL transition) is 44 (88-44) kJ mol^{-1}. Thus, the presence of the HS defect at Fe(2) stabilises the HS state at Fe(1) at 11 kJ mol^{-1}. On the other hand, for the HS matrix of **1** the spin switching of the LS Fe(1) is 33 kJ mol^{-1}. With another LS defect at Fe(2) (HLLHH isomer) the spin switching energy (i.e. formation of HLHHH) is 64 kJ mol^{-1} (i.e 178 -114). Thus, the presence of the LS defect at Fe(2) stabilises the LS state at Fe(1) at 31 kJ mol^{-1}.

H_{2D} – Here, we consider the effect of the two defects nearest to Fe(1), i.e. the switching from LHLHL to LHHHL and HLLLH to HLHLH, compared to creation/annihilation of single defect at Fe(1) (i.e. formation of LLHLL from LLLLL or HHHHH from HHLHH). For the low-spin matrix of **1** (in this case defined by the spin states of both Fe(3) centres) the spin switching energy of the LHLHL → LHHHL transition is 35 kJ mol^{-1} compared to 55 kJ mol^{-1} for the LLLLL → LLHLL transition. Thus, the presence of two HS defects at Fe(2) and Fe(2') stabilises the HS state at Fe(1) at 20 kJ mol^{-1}. The analogous effect for the HS matrix of **1** is only 2 kJ mol^{-1}.

H_{N2D} – This parameter describes how much is the energy of the LS → HS transition of Fe(1) dependent on the spin states of both Fe(3) and Fe(3') centres. In other words, we look at the next-to-nearest neighbours effect on the spin transition of a Fe(II) centre. Namely, we consider the HLLLH → HLHLH and LHLHL → LHHHL transition and compared their electronic energies with that of LLLLL → LLHLL and HHLHH → HHHHH, respectively. For the low-spin matrix of **1** the spin switching energy of the HLLLH → HLHLH transition is 39 kJ mol^{-1} compared to 55 kJ mol^{-1} for the LLLLL → LLHLL transition. Thus, the presence of two HS defects at Fe(3) and Fe(3') stabilises the HS state at Fe(1) at 16 kJ mol^{-1}. The analogous effect for the HS Fe(2)-Fe(1)-Fe(2') chain/inner matrix is of **1** only 2 kJ mol^{-1}. It is to note that for **2a** the effect for the HS is higher than for the LS one.

H_{ND} - This parameter describes how much is the energy of the LS → HS transition of Fe(1) dependent on the spin states of Fe(3) centre. Namely, we consider the LLLLL → LLHLL and HLLLL → HLHLL transition and compared their electronic energies with that of LLLLL → LLHLL and HHLHH → HHHHH, respectively. For the low-spin matrix of **1** the spin switching energy of the HLLLL → HLHLL transition is 54 kJ mol^{-1} compared to 55 kJ mol^{-1} for the LLLLL → LLHLL transition. On the other hand, there is a large energy difference between the energies of the LHLHH to LHHHH and HHLHH to HHHHH transitions (63 and 33 kJ mol^{-1}, respectively).

H_{3D} – The parameter that describes the effect of the spin of the centre separated with two others from the switching one. Namely, we consider the energies of the LLLLL → LHLLL and the HLLLL → LHHLH transitions (LS matrix) and that of HLHHL → HHHHL and HLHHL → HLHHH transitions (HS matrix). While the first two differ only at 0.5 kJ mol^{-1} for **1** the corresponding to a switching in a HS matrix differ at 22 kJ mol^{-1}.

H_{4D} – In this case, we compare how the spin of the Fe(3) centre influences the LS→HS transition of the Fe(3') one with three iron centres separating them (Fe(2), Fe(1) and Fe(2') stay in either LS on HS state, defining the matrix).

S4. Dependence of H_{coop} on the applied DFT tools.

Table S2. H_{coop} (kJ ·mol^{-1}) for the systems **1**, **2** and **2a** optimised with the different exchange-correlation functionals and basis sets. The calculations with B3LYP*, B3LYP and CAM-B3LYP were performed with the CEP-31G functional, those with TPSS and TPPSh with the tzvp basis sets. For B3LYP, CAM-B3LYP and TPSS calculations the Grimme's D3 dispersion correction was used. The results show a fairly consistent pattern, particularly within the series of B3LYP type functionals.

	**1**	**2**	**2a**
B3LYP/D3	**25**	**14**	**26**
B3LYP	24	14	
CAM-B3LYP	23	13	
CAM-B3LYP/D3	24	19	25
B3LYP*	**22**	19.5	28
PBEh	**24**		
TPSS/D3	**14**	17	
TPSSh	**16**	16	

Table S3. Calculated electronic energies and H_{strain} parameters obtained with B3LYP/D3 CAM-B3LYP/D3 and PBEh functionals for **2**.

	B3LYP/D3		CAM-B3LYP/D3		PBEh	
	E_{el}	H_{strain}	E_{el}	H_{strain}	E_{el}	H_{strain}
**LLLLL**	0		19		0	
**LLHLL**	25	2	37	2		
**LHLLL**	44	20	29	20	5	19
**HLLLL**	34	11	67	13	-2	11
**HLLHL**	78	32	37	33	3	17
**LHHLL**	52	6	48	4	-14	13
**LLHLH**	59	12	59	14	-16	11
**HLLLH**	69	23	73	25	-5	22
**LHLHL**	86	40	56	39	10	37
**LLLHH**	66	20	55	22	-7	19
**HLLHH**	93	24	62	5	-37	3
**HLHHL**	83	13	76	12	-21	19
**HLHLH**	92	23	58	26	-20	20
**LHHHL**	80	11	92	8	-20	20
**LHLHH**	109	39	51	41	-3	37
**LLHHH**	70	0	78	0	-40	0
**HHLHH**	105	12	83	10	-39	15
**HLHHH**	109	16	78	16	-45	9
**LHHHH**	105	12	84	10	-44	10
**HHHHH**	116		19		-67	
H_{coop}	14		12.5			
E_{ad}	23		17		-13	

Table S4. Calculated electronic energies and H_{strain} parameters (kJ ·mol^{-1}) obtained with B3LYP/D3 for **2a.** Zero-point corrections are given. The ZPE and H_{strain} values are also given for B3LYP*.

	B3LYP/D3	B3LYP/D3	B3LYP*	B3LYP/D3	B3LYP*
	E_{el}	ZPE	ZPE	H_{strain}	H_{strain}
**LLLLL**	0	**0**	**0**		
**LLHLL**	50	**-14**	**-14**	**17**	**18**
**LHLLL**	41	**-13**	**-13**	**7**	**8**
**HLLLL**	45	**-11**	**-12**	**11**	**11**
**HLLHL**	85	**-24**	**-25**	**18**	**18**
**LHHLL**	76	**-27**	**-26**	**9**	**12**
**LLHLH**	95	**-26**	**-26**	**28**	**28**
**HLLLH**	89	**-23**	**-24**	**22**	**21**
**LHLHL**	80	**-25**	**-24**	**13**	**13**
**LLLHH**	73	**-24**	**-25**	**6**	**6**
**HLLHH**	117	**-35**	**-37**	**16**	**17**
**HLHHL**	120	**-38**	**-38**	**20**	**21**
**HLHLH**	140	**-38**	**-38**	**40**	**37**
**LHHHL**	103	**-39**	**-35**	**2**	**15**
**LHLHH**	112	**-37**	**-36**	**11**	**12**
**LLHHH**	109	**-38**	**-38**	**9**	**10**
**HHLHH**	143	**-48**	**-48**	**9**	**10**
**HLHHH**	153	**-49**	**-50**	**19**	**19**
**LHHHH**	134	**-50**	**-50**	**0**	**2**
**HHHHH**	168	**-62**	**-62**		
E_{ad}	34				
H_{coop}	25				

Table S5 Calculated electronic energies (kJ·mol^{-1}, B3LYP/D3), zero-point energy differences and H_{strain} (kJ·mol^{-1}) for **3** relative to those for LLLLL isomer.

	B3LYP/D3		
	E_{el}	ZPE	H_{strain}
**LLLLL**	0	**0**	
**LLHLL**	29	**-11**	
**LHLLL**	11	**-11**	**7**
**HLLLL**	23	**-12**	**11**
**HLLHL**	45	**-23**	**18**
**LHHLL**	41	**-22**	**9**
**LLHLH**	39	**-23**	**28**
**HLLLH**	27	**-23**	**22**
**LHLHL**	61	**-20**	**13**
**LLLHH**	40	**-24**	**6**
**HLLHH**	72	**-34**	**16**
**HLHHL**	65	**-34**	**20**
**HLHLH**	51	**-34**	**40**
**LHHHL**	64	**-32**	**2**
**LHLHH**	89	**-34**	**11**
**LLHHH**	74	**-34**	**9**
**HHLHH**	81	**-46**	**9**
**HLHHH**	63	**-43**	**19**
**LHHHH**	71	**-45**	**0**
**HHHHH**	91	**-56**	
E_{ad}	18		
H_{coop}	33		

Table S6. Zero-point energy differences (kJ·mol^{-1}) for **1** relative to those for LLLLL isomer calculated with B3LYP and CAM-B3LYP with and without dispersion corrections.

	CAM-B3LYP/D3	CAM-B3LYP	B3LYP/D3	B3LYP
**LLLLL**	**0**	**0**	**0**	**0**
**LLHLL**	**-12**	**-12**	**-12**	**-12**
**LHLLL**	**-12**	**-11**	**-12**	**-11**
**HLLLL**	**-12**	**-12**	**-12**	**-12**
**HLLHL**	**-24**	**-23**	**-24**	**-23**
**LHHLL**	**-24**	**-23**	**-24**	**-23**
**LLHLH**	**-24**	**-23**	**-24**	**-22**
**HLLLH**	**-24**	**-23**	**-25**	**-23**
**LHLHL**	**-23**	**-29**	**-23**	**-22**
**LLLHH**	**-24**	**-23**	**-24**	**-23**
**HLLHH**	**-36**	**-35**	**-36**	**-34**
**HLHHL**	**-35**	**-34**	**-36**	**-34**
**HLHLH**	**-36**	**-34**	**-36**	**-34**
**LHHHL**	**-35**	**-34**	**-36**	**-33**
**LHLHH**	**-35**	**-34**	**-36**	**-34**
**LLHHH**	**-36**	**-34**	**-36**	**-34**
**HHLHH**	**-48**	**-46**	**-48**	**-45**
**HLHHH**	**-48**	**-46**	**-48**	**-45**
**LHHHH**	**-48**	**-46**	**-48**	**-45**
**HHHHH**	**-60**	**-57**	**-60**	**-56**

			Fe(3)	Fe(2)	Fe(1)	Fe(2')	Fe(3')
**1**	B3LYP*	LLLLL	1.995/*1.999*	1.985/*1.989*	1.979/*1.989*		
	B3LYP/D3		1.990/1.995	1.990/*1.986*	1.985/*1.986*		
	PBEh		1.976/*1.982*	1.975/*1.974*	1.965/*1.973*		
	B3LYP*	HHHHH	2.225/***2.134***	2.225/***2.131***	2.205/***2.132***		

	B3LYP/D3		2.198/***2.121***	2.197/***2.120***	2.204/***2.118***		
	PBEh		2.196/2.119	2.197/*2.124*	2.203/*2.121*		
	B3LYP*	LLHLL	1.992/1.996	**2.000**/*1.998*	**2.175**/*2.119*		
	B3LYP/D3		1.991/1.995	**1.988**/*1.994*	**2.162**/*2.110*		
	PBEh		1.977/*1.983*	1.984/*1.982*	2.162/2.112		
**2a**	B3LYP*	LLLLL	1.983	1.989	1.978		
	B3LYP/D3		1.983	1.988	1.978		
	B3LYP*	HHHHH	**2.167**	**2.180**	2.176	check	
	B3LYP/D3		**2.150**	**2.165**	2.164		
	B3LYP*	HLHLH	2.152	2.012	2.144		
	B3LYP/D3		2.154	2.012	2.144		
**3**	B3LYP*	LLLLL	2.001	1.994	1.991	1.986	1.996
	B3LYP/D3		1.997	1.986	1.986	1.983	1.989
	B3LYP*	HHHHH	**2.189**	**2.177**	**2.186**	**2.183**	**2.182**
	B3LYP/D3		**2.163**	**2.161**	**2.167**	**2.162**	**2.171**
	B3LYP*	LHLHL	2.000	**2.159**	2.009	**2.162**	2.008
	B3LYP/D3		1.995	**2.141**	2.006	**2.140**	2.003

Table S7. Comparison of the mean Fe-N values for the LLLLL and HHHHH and the spin isomers revealing the largest strain obtained for the complexes under study for **2a** (very close values of H_{strain} for calculations with B3LYP* and B3LYP/D3) and **1** and **3** (larger discrepancy of respective H_{strain} values). The distances for the negatively charged trz$^-$ ligand in **1** are given in italics. The values that differ at more 0.01 Å are marked bold. Note that that large differences concern nearly exclusively the bond-lengths of the HS centre. It is to observe that there is a systematic shortening of the calculated Fe-N bonds for HHHHH isomer on going from B3LYP* to B3LYP, the corresponding distances for the HLHLH match perfectly for both spin isomers. This not the case for **3**, displaying the largest discrepancies of H_{strain} for both functionals.

S5. Vibrational entropy as function of the applied DFT functional.

It is of importance to assess how much is the vibrational entropy dependent on the choice of functional. Therefore, we performed the calculations of the normal vibrations for model systems **1**-**3** after optimisation with B3LYP/D3 method to compare the obtained S_{vib}(T) dependencies with those calculated for the B3LYP* functional. The results are shown in Figure S15. The first inspection reveals that the obtained patterns are by and large similar for both functionals, showing a "packet-like" dependence of entropy curves on the number of the HS centres for **1** with the S_{vib}(T) curves for the given group of L^nH^{5-n} spin isomers with more spread values for **2** and **3**. For **2a**, the calculations with B3LYP/D3 yield the „packet-like" pattern, while B3LYP* yields more spread values. The comparison of the ranges of the S_{vib} values at 300 K is summarised in Table S8 below.

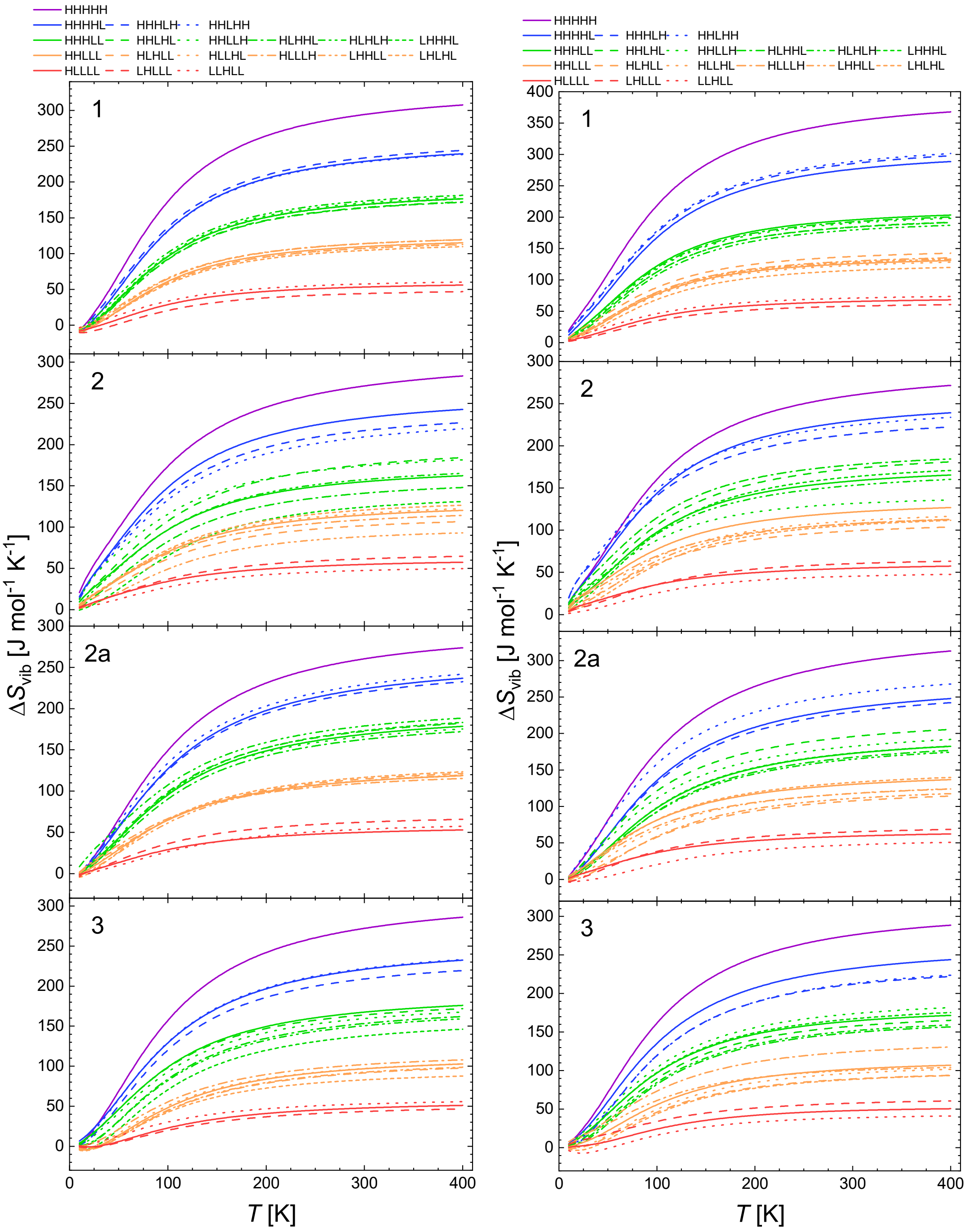

Figure S15. Comparison of the calculated temperature dependence of vibrational entropy calculations of the spin transition from the LLLLL spin isomers for the model systems optimised with B3LYP/D3 (left) and B3LYP* (right).

	L^4H^1		L^3H^2		L^2H^3		L^1H^4		H^5	
	B3LYP/D3	B3LYP*	B3LYP/D3	B3LYP*	B3LYP/D3	B3LYP*	B3LYP/D3	B3LYP*	B3LYP/D3	B3LYP*
**1**	43-56	57-73	103-114	117-138	162-175	178-199	227-233	275-292	295	355
**2**	47-61	45-61	88-120	99-121	125-175	132-178	208-231	213-230	271	259
**2a**	50-63	46-67	109-118	107-134	163-180	164-197	219-228	227-255	261	298
**3**	43-52	39-59	82-101	88-125	139-170	148-175	208-222	213-234	271	277

Table S8. Vibrational entropy (J· K^{-1} ·mol^{-1}) at 300 K ranges for different spin isomers containing the same amount of HS centres for the modelled systems.

The analysis of the data in Table S8 allows the following conclusions to be drawn.

(i) The largest discrepancy occurs for the full HS isomer HHHHH of 1 yielding the difference between B3LYP/D3 and B3LYP* functionals to be 60 entropy units. This effect may be related to the discussed differences in the Fe-N distances obtained with the two methods for 1. The differences smaller for the two other systems.

(ii) for the L^1H^4 the difference between the results of the two functionals are again the largest for 1 with the results for 2 and 3 lying in the comparable ranges.

(iii) for the other three groups of spin isomers the difference becomes smaller, the values for 1 still larger for B3LYP/D3 than for B3LYP*.

In the next step, we compared the entropy values obtained with B3LYP/D3, CAM-B3LYP/D3 and with both these functionals without the dispersion corrections.

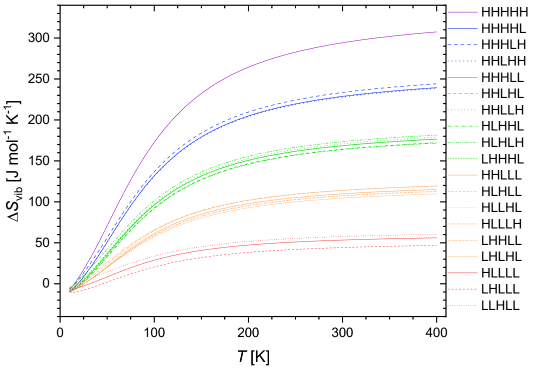

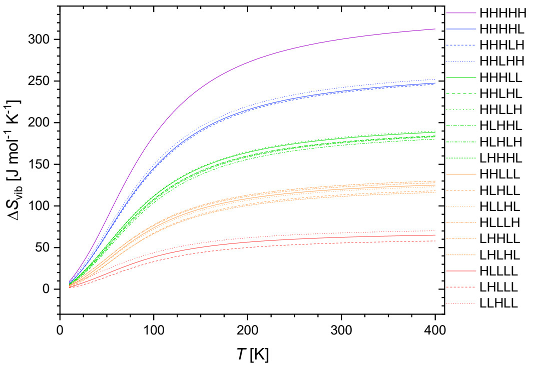

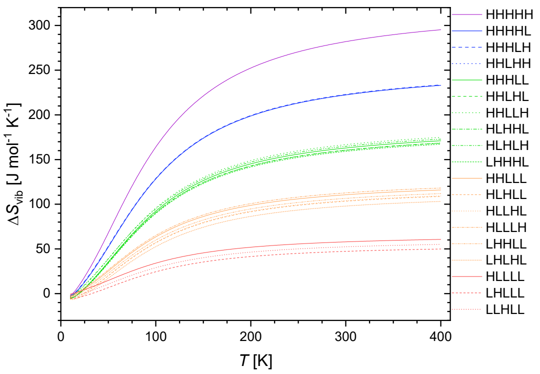

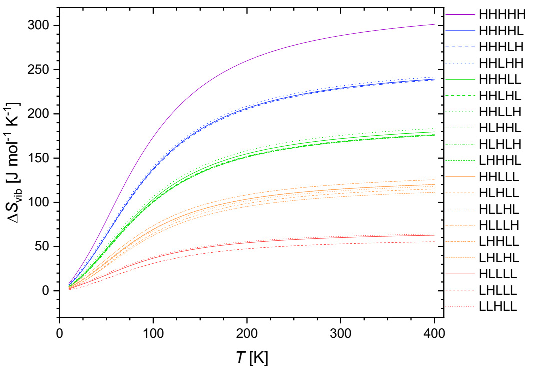

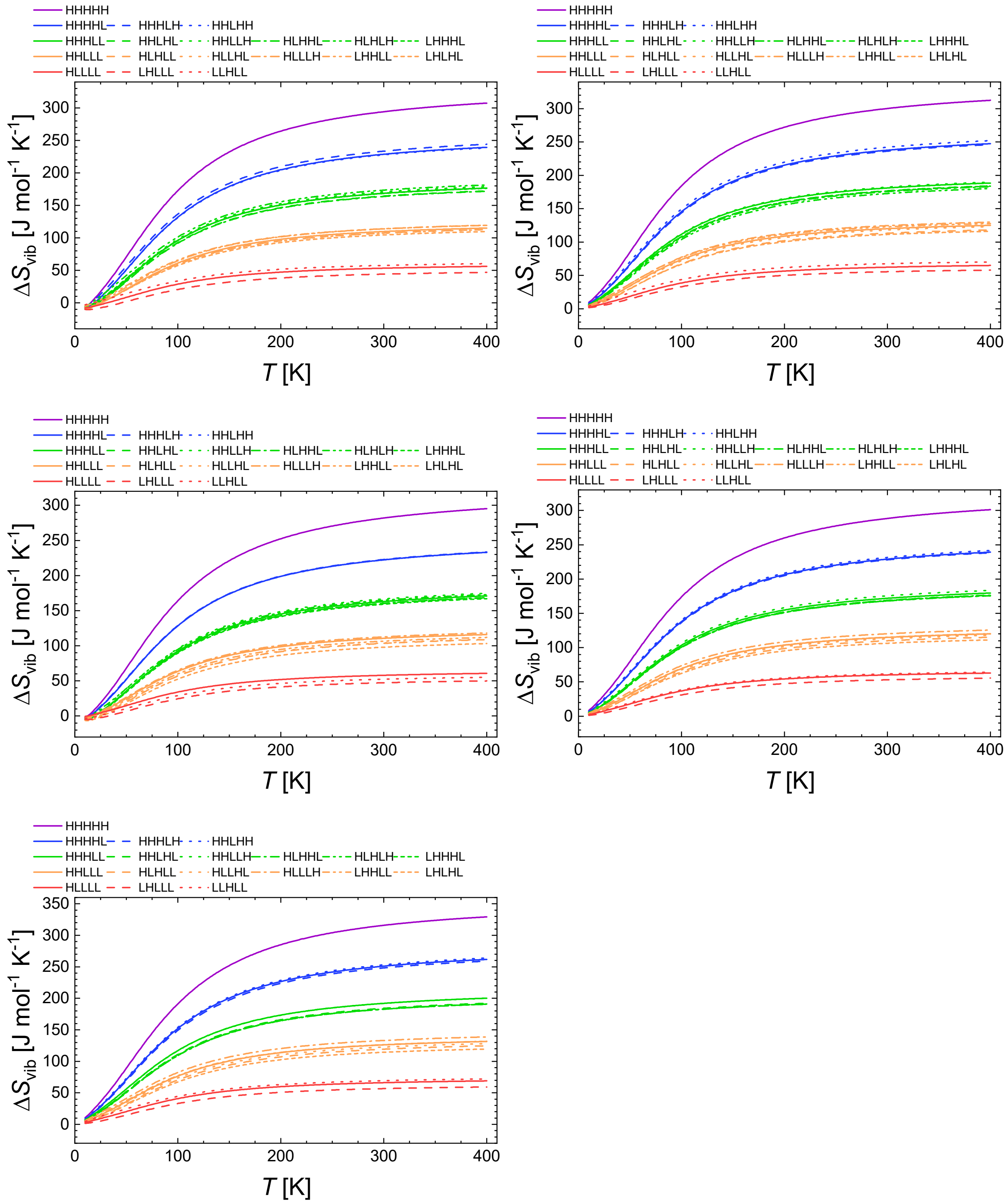

Figure S16. Comparison of the calculated temperature dependence of vibrational entropy calculations of the spin transition from the LLLLL spin isomers for **1** optimised with B3LYP/D3 (top left), B3LYP (top right), CAM-B3LYP/D3 (middle, left), CAM-B3LYP (middle, right), PBEh (bottom, left).

These results show a quite satisfactory matching of the four approaches. The S_{vib}(300K) for the LLLLL to HHHHH transition is 295, 302, 282 and 287 J· K^{-1} ·mol^{-1} for B3LYP/D3, B3LYP, CAM-B3LYP/D3 and CAM-B3LYP, respectively. The mean entropies of L^4H^1 spin isomers calculated for 300K are, respectively 50, 62, 53 and 56 J· K^{-1} ·mol^{-1}. The value for PBEh for the LLLLL to HHHHH transition is 319 J· K^{-1} ·mol^{-1} and the mean value for L^4H^1 spin isomers calculated for 300 K is 63 J· K^{-1} ·mol^{-1}.

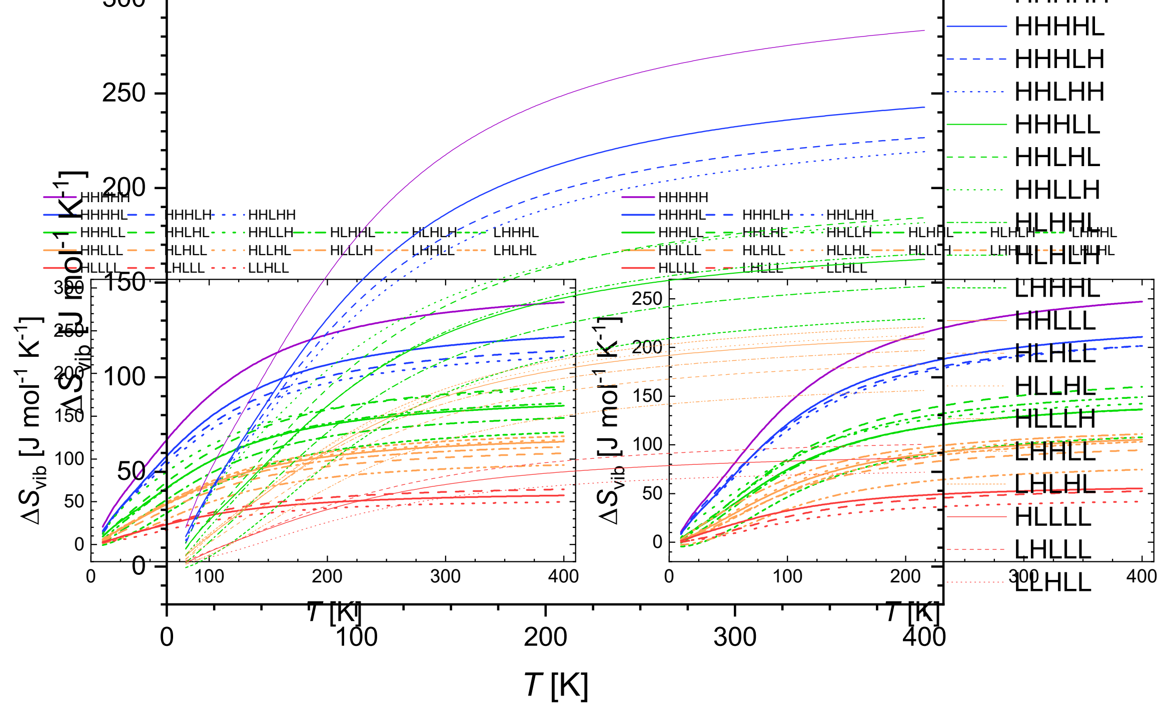

Figure S17. Comparison of the calculated temperature dependence of vibrational entropy calculations of the spin transition from the LLLLL spin isomers for the model system **2** optimised with B3LYP/D3 (left), and CAM-B3LYP/D3 (right)

Finally, we compared the results of the calculated S_{vib}(T) dependencies for **2** calculated with B3LYP/D3 and CAM-B3LYP/D3 (see Figure S19). The S_{vib}(300K) for the LLLLL to HHHHH transition is 271 and 236 J· K^{-1} ·mol^{-1} for B3LYP/D3 and CAM-B3LYP/D3, respectively, while the mean entropies of L^4H^1 spin isomers calculated for 300 K are, respectively, 54 and 45 J· K^{-1} ·mol^{-1}

S7.

1

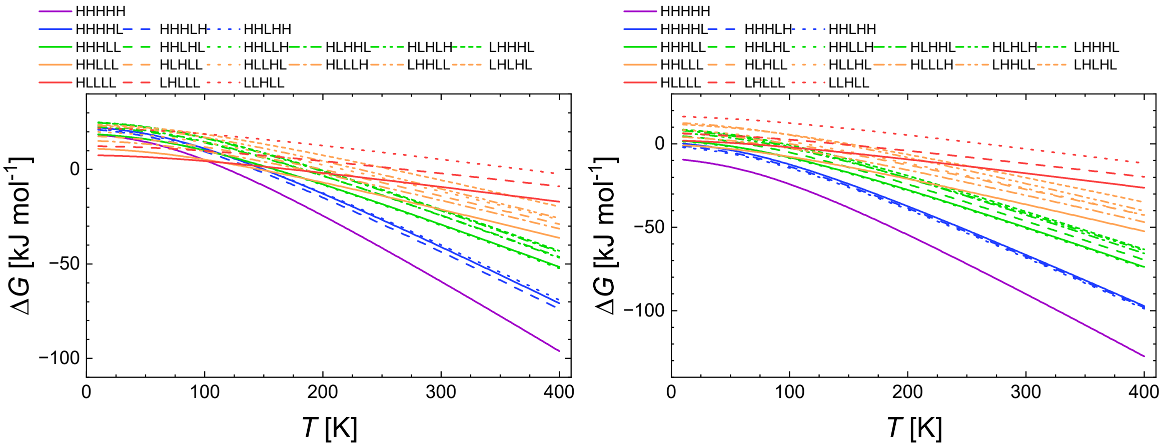

B3LYP-D3 B3LYP-nodisp

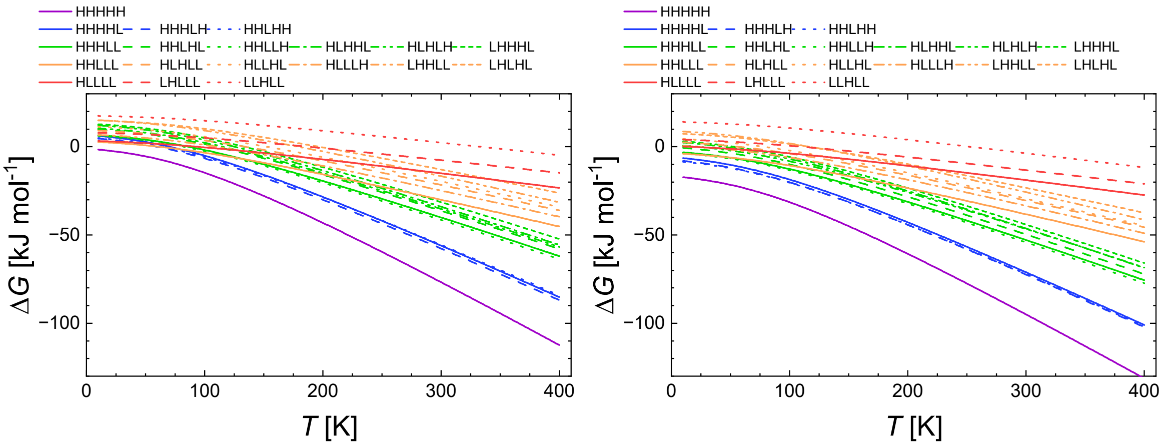

CAM-B3LYP CAM-nodisp

Figure S18. Temperature dependence of ΔG of the LLLLL to HHHHH transition for **1**. Top: calculated with B3LYP/D3 (left) and without dispersion correction (right). Bottom: calculated with CAM-B3LYP/D3 (left) and without dispersion correction

2

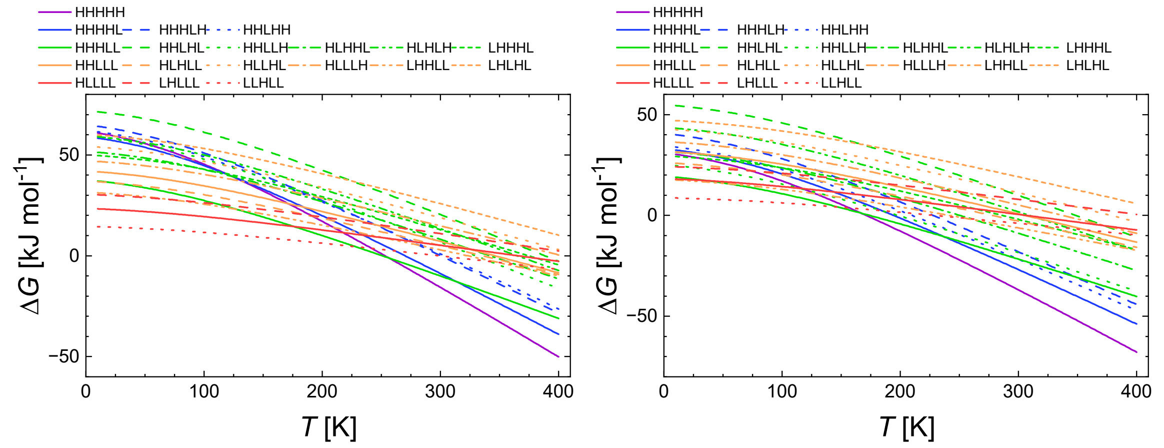

B3LYP-D3 CAM-B3LYP

Figure S19. Temperature dependence of ΔG of the LLLLL to HHHHH transition for **2**. Left: calculated with B3LYP/D3 (left). Rights: calculated with CAM-B3LYP/D3 (left).

S8. Remarks on the completeness of spin transition for the calorimetrically measured samples of $[Fe(NH_2trz)_3]Cl_2$ and ($[Fe(NH_2trz)_3]Cl_2{\cdot}2H_2O$).

Due to the exceptionally high spin transition temperatures of complexes **2** and **2a** and the potential dehydration of the hydrated form, no Mössbauer data are available at high temperatures, making practical measurements in the fully high-spin state unfeasible.

To address this limitation, we refer to magnetochemical studies and Nuclear Inelastic Scattering (NIS) measurements on similar $[Fe(NH_2trz)_3]Cl_2$-type compounds reported by Lavrenova *et al.*[1] and Rackwitz *et al.*.[2] NIS studies show that the vibrational modes of $[Fe(NH_2trz)_3]Cl_2$ at room temperature are nearly identical to those at 100 K, and no significant spectral changes are observed upon heating. This supports the conclusion that no spin transition occurs between 100 K and 300 K, consistent with the reported high $T_{1/2}$ value of ~355 K. Furthermore, magnetic data (μ_{eff}-T curves) for both the hydrated ($[Fe(NH_2trz)_3]Cl_2{\cdot}2H_2O$) and dehydrated ($[Fe(NH_2trz)_3]Cl_2$) forms reveal step-like increases in μ_{eff} with increasing temperature, indicative of spin transition behavior. However, neither system reaches a fully HS state. In particular, the hydrated compound

retains a significant LS fraction even at high temperatures. The dehydrated form exhibits a more complete spin transition and a higher $T_{1/2}$, approximately 10 K above that of the hydrated form. These trends are consistent with our DSC results, where the heating transition temperatures are 332 K for **2a** and 343 K for **2**, respectively.

The values obtained from DSC should be interpreted with caution, as they reflect an incomplete spin transition. In addition, they may be influenced by chain-end effects, lattice defects, and limited structural cooperativity. These factors contribute to the discrepancy between the measured values and the ideal values expected for a fully cooperative, 100% spin transition.

1. Lavrenova, L.G., Shakirova, O.G., Ikorskii, V.N., Varnek, V.A., Sheludyakova, L.A. and Larionov, S.V., $^1A_1 \rightleftarrows {}^5T_2$ Spin Transition in New Thermochromic Iron (II) Complexes with 1, 2, 4-Triazole and 4-Amino-1, 2, 4-Triazole. *Russ. J. Coord. Chem.* **2003**, *29*, 22-27.
2. Rackwitz, S.; Wolny, J. A.; Muffler, K.; Achterhold, K.; Rüffer, R.; Garcia, Y.; Diller, R.; Schünemann, V., Vibrational properties of the polymeric spin crossover (SCO) Fe(II) complexes [{Fe(4-amino-1, 2, 4-triazole)$_3$}X_2]*n*: a nuclear inelastic scattering (NIS), Raman and DFT study. *Phys. Chem. Chem. Phys.* **2012**, *14*, 14650-14660.
